# Frailty and long-term outcomes in younger patients with acute myocardial infarction

**DOI:** 10.1093/eurheartj/ehaf876

**Published:** 2026-06-02

**Authors:** Hasan Mohiaddin, Shirley Sze, Abdulla A. Damluji, Andrew Ladwiniec, Gerry P. McCann, Gavin J. Murphy, Stefan James, Chris P. Gale, Iain Squire, Muhammad Shahzeb Khan, Javed Butler, Mamas A. Mamas, Muhammad Rashid

**Affiliations:** 1 NIHR Biomedical Research Centre, University of Leicester, Glenfield Hospital, Groby Road, Leicester LE3 9QP, UK; 2 Keele Cardiovascular Research Group, Centre for Prognosis Research, Keele University, Keele Rd, Stoke-on-Trent ST5 5BG, UK; 3 Inova Center of Outcomes Research, Inova Heart and Vascular Institute, Falls Church, VA, USA; 4 Division of Cardiology, Department of Medicine, Johns Hopkins University School of Medicine, Baltimore, MD, USA; 5 Department of Cardiovascular Sciences, University of Leicester, Glenfield Hospital, Groby Road, Leicester LE3 9QP, UK; 6 Department of Medical Sciences, Cardiology and Uppsala Clinical Research Center, Uppsala University, Uppsala 751 85, Sweden; 7 Leeds Institute of Cardiovascular and Metabolic Medicine, University of Leeds, Leeds, UK; 8 Department of Cardiology, Leeds Teaching Hospitals NHS Trust, Leeds, UK; 9 Baylor Scott and White Research Institute, Dallas, TX, USA; 10 Department of Medicine, University of Mississippi, Jackson, MS, USA

**Keywords:** Acute myocardial infarction, Frailty, Mortality, Quality of care

## Abstract

**Background and Aims:**

Frailty is increasingly recognized as an important determinant of adverse outcomes in older adults with acute myocardial infarction (AMI), but its impact in younger patients remains underexplored. The aims of this study were to evaluate the association of frailty with adverse outcomes in AMI patients, stratified by age.

**Methods:**

This population-based epidemiological study utilized linked national administrative data from England and Wales. Patients were stratified into three age groups: <55 year, 55–74 years, and ≥75 years. Frailty was assessed using the Secondary Care Administrative Records Frailty index with patients categorized into fit, mild, moderate, and severe groups. All-cause mortality at 1 year was the primary outcome. Secondary outcomes were cardiovascular and bleeding-related events.

**Results:**

A total of 931 133 patients were included of which 13% of patients were severely frail. In patients with severe frailty, adjusted hazard ratios for all-cause mortality were 6.69 [95% confidence interval (CI) 5.76–7.76] for young patients, 4.33 (95% CI 4.11–4.57) for middle-aged patients, and 2.31 (95% CI 2.23–2.39) for older patients. The interaction between age and frailty revealed that younger patients with severe frailty had a 3.51-fold (95% CI 3.11–3.96) higher risk of all-cause mortality compared with older patients with severe frailty (*P* < .001).

**Conclusions:**

Frailty is independently associated with poor outcomes after AMI, with the strongest impact observed in younger patients, highlighting the need for frailty assessment across all age groups.

## Structured Graphical Abstract

**Key Question:**

Is the impact of frailty on mortality modified by age in patients with acute myocardial infarction (AMI)?

**Key Finding:**

Among 931 133 patients, frailty was associated with up to a 3.5x greater relative risk of all-cause mortality in young patients (<55 years old) when compared to older patients (≥75 years old).

**Take Home Message:**

Frailty is a prevalent and critical risk factor in adults with AMI of all ages. However, its impact is more profound in young patients, underscoring a need for optimised methods of frailty assessment and targeted treatment strategies.

Pictorial representation of the total number of patients with acute myocardial infarction in each age and frailty group. An interaction analysis between age and frailty categories revealed that across all frailty strata, frailty in young patients was associated with a greater relative risk of all-cause mortality when compared with older patients.

## Graphical Abstract

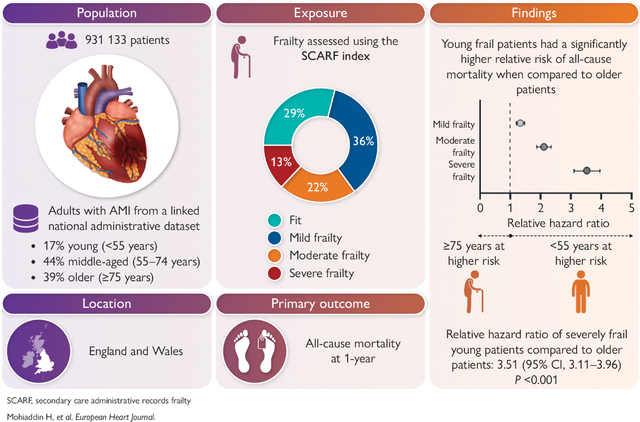

## Introduction

Acute myocardial infarction (AMI) remains a leading cause of cardiovascular mortality and morbidity in the young.^1^ Frailty, a state of increased vulnerability to stressors, is increasingly recognized as an important factor influencing outcomes after AMI.^2–4^ Frailty is a multi-dimensional syndrome encompassing medical comorbidities, increased dependence, social vulnerability, nutritional problems, and cognitive decline, leaving individuals susceptible to adverse health events.^5,6^ Large-scale studies have demonstrated that frailty is not confined to older adults.^7–9^ A UK biobank study has shown that frailty is prevalent in individuals aged 37–73 years old with a clear association identified between frailty and increased mortality, independent of chronological age.^7^ Furthermore, frailty is strongly associated with all-cause and cause-specific mortality in adults as young as 30 years old, with younger people living with frailty identified as a particularly high-risk population.^8^ These findings underscore the necessity of addressing frailty across the entire age spectrum in patients with AMI, as younger frail patients may represent a high-risk subgroup requiring tailored interventions. Despite growing evidence of its significance, the age-specific prevalence and impact of frailty in younger AMI patients remain underexplored.

Our objectives were to determine the prevalence of frailty across different age groups in patients admitted with AMI and to assess whether there is an interaction between age and frailty which influences long-term (1-year) outcomes.

## Methods

### Data sources

The Myocardial Ischaemia National Audit Project (MINAP) is a national AMI registry, collecting data on patients hospitalized with AMI in England and Wales, including demographics, clinical characteristics, treatments, in-hospital outcomes, and discharge information.^10^ Hospital Episode Statistics Admitted Patient Care (HES-APC) contains International Classification of Diseases 10th Edition (ICD-10) coded data on all admissions to National Health Service (NHS) hospitals in England. Office of National Statistics (ONS) mortality data contains ICD-10 coded records on deaths in the UK.^11^ This study used a linked multi-source dataset of MINAP, HES-APC, and ONS mortality data.

Formal ethical approval for the linkage of MINAP, HES-APC, and ONS registries was granted by the Health and Care Research Wales and the Health Research Authority (Research Ethics Committee reference 20/WA/0312).^12^ Additional approval was obtained by the Confidentiality Advisory Group, an independent committee that advises on the use of confidential patient information for research.^13^ This work was conducted in compliance with STROBE guidelines for observational studies and CODE-EHR best practice framework for the use of electronic healthcare records in clinical research.^14,15^

### Study population

We included adults (aged ≥18 years) in MINAP admitted to hospitals between 1 January 2005 and 31 March 2019 with a final diagnosis of AMI. Diagnosis of AMI was determined locally by clinicians according to patient symptoms, clinical examination, and in-patient investigations as per the consensus document of the European Society of Cardiology (ESC) and American College of Cardiology.^16^ Patients with missing data on age, final diagnosis, and date of discharge were excluded. In patients with multiple MINAP appearances in a single calendar year, the first AMI admission was considered. To determine the differential effect of frailty on older vs younger patients, we stratified the overall cohort into young (age <55 years), middle-aged (55–74 years), and older groups (≥75 years).^17^

### Frailty index

The Secondary Care Administrative Records Frailty (SCARF) index, a validated cumulative deficit frailty index (FI) encompassing 31 health deficit domains derived from HES-APC ICD-10 codes, was used to measure frailty in our cohort.^18^ The SCARF index has demonstrated strong predictive validity for adverse outcomes in prior studies.^19^ Deficit domains included impairment of function, nutritional and cognitive problems, geriatric syndromes, social vulnerability, and medical co-morbidities. Patients were then grouped according to four levels of frailty depending on the total number of deficits they accumulated: fit (FI = 0–0.05 or 0–1 deficit), mild frailty (FI = 0.06–0.11 or 2–3 deficits), moderate frailty (FI = 0.12–0.18 or 4–5 deficits), and severe frailty (FI ≥ 0.19 or ≥6 deficits).^18^ To prevent overestimation of frailty in our AMI cohort, patients were only given a point for ischaemic heart disease in SCARF if they had a prior history of myocardial infarction, angina, percutaneous coronary intervention (PCI), or coronary artery bypass graft (CABG) procedures.

The Charlson comorbidity index (CCI) was derived in a similar manner.^20^ Further details on the HES-APC ICD-10 codes and MINAP variables used to calculate SCARF and CCI are summarized in Supplementary data online, Table S1.^18^

### European Society of Cardiology acute myocardial infarction quality of care indicators

Adherence to ESC AMI quality of care indicators were determined from data in MINAP and included door-to-balloon time (DTBT) in patients with ST-elevation myocardial infarction (STEMI), time to angiography in patients with non-ST-elevation myocardial infarction (NSTEMI), admission location (excluding admissions to intensive care), referral for cardiac rehabilitation on discharge, and prescription of guideline-recommended secondary prevention medications.^21^ The opportunity-based quality indicator (OBQI) score, detailing overall adherence to evidence-based processes of care, was determined. This included the prescription of aspirin, a second antiplatelet agent, beta-blockers, angiotensin-converting enzyme inhibitors (ACEis) or angiotensin receptor blockers (ARBs), statins, and enrolment to a cardiac rehabilitation programme at the time of discharge.^21^

### Outcomes

The primary outcome was all-cause mortality at 1 year. Secondary outcomes included cardiovascular mortality, major adverse cardiovascular events (MACE), reinfarction, heart failure (HF) readmission, major bleeding, and minor bleeding. Time to event for mortality and hospital readmissions from discharge were determined using ONS mortality and HES-APC data, respectively, using the ICD-10 codes described in Supplementary data online, Table S2. MACE were defined as a composite of all-cause death or rehospitalization due to ischaemic stroke, reinfarction, or HF.^22^ Cardiovascular mortality was defined as any death caused by ischaemic stroke, AMI, or HF. Major bleeding included any fatal bleed or hospital admission related to gastrointestinal or neurological haemorrhage or ruptured aneurysm. Minor bleeding included ICD-10 diagnoses unlikely to require urgent medical intervention. A sensitivity analysis of short-term outcomes (up to 30 days) was performed including in-hospital events from MINAP and ONS datasets. Major in-hospital bleeding was defined as intracranial or retroperitoneal haemorrhage, or any bleed with a fall in haemoglobin of ≥3 g/dL. Minor in-hospital bleeding was defined as any bleed with a fall in haemoglobin of <3 g/dL. In-hospital reinfarction was determined according to clinical symptoms, new electrocardiographic changes, and elevation of any biomarker of cardiac necrosis beyond the upper normal limit or ≥50% of the last recorded value.^23^

### Statistical analysis

Clinical data were compared across frailty groups in the entire AMI cohort and between age groups. Continuous variables are presented as median and interquartile range (IQR) and categorical data as counts and percentages. Years of life lost (YLL) were calculated for patients who died within 1 year of AMI admission using UK life expectancy tables, available on the ONS website.^24^

Missing data were addressed using multiple imputations with chained equations, assuming ‘missingness’ occurred at random.^25^ Ten imputed datasets were generated. Multivariate Cox regression analyses were conducted on each imputed dataset and combined using Rubin’s rules.^26^ Adjusted hazard ratios (aHRs) with 95% confidence intervals (CIs) were determined for the time to each outcome within 1 year of the index admission for each patient age group using fit patients as a reference. Covariates included year of admission, age, sex, ethnicity, smoking, creatinine, left ventricular (LV) function, Killip class, ST-segment elevation, elevation of biomarkers of myocardial injury, hypercholesterolaemia, cardiac arrest, family history of coronary artery disease (CAD), in-patient revascularization (with PCI or CABG), in-hospital pharmacotherapy, and referral for cardiac rehabilitation on discharge. Medications included beta-blockers, ACEis or ARBs, mineralocorticoid receptor antagonists, glycoprotein IIb/IIIa antagonists, fondaparinux or low molecular weight heparin, unfractionated heparin, warfarin, dual antiplatelets, and statins. We then examined the association between frailty status and age groups (<55 years and 55–74 years compared with ≥75 years) with outcomes of interest. Cox proportional hazards models were used, incorporating interaction terms between frailty status and age categories to assess potential effect modification. Relative hazard ratios (rHRs) with 95% CIs were reported, along with *P*-interaction values to evaluate the statistical significance of the interaction terms. The model adjusted for relevant clinical and demographic covariates, including those listed above. Proportional hazard assumptions were assessed using Schoenfeld residuals, and statistical significance was defined as a two-tailed *P* < .05. The interaction between age (as a continuous variable in years) and frailty (using the continuous SCARF index score) was also assessed using a Cox proportional hazards model, including an interaction term for age × frailty score.

For non-fatal time-to-event outcomes, death was considered a competing risk. We used standard cause-specific Cox proportional hazards models, which estimate the instantaneous rate of a specific event (e.g. reinfarction) in subjects who are currently alive and have not yet experienced that event. This is a valid and commonly used approach for estimating the direct aetiological effect of covariates on the cause-specific hazard in the presence of competing risks. To assess for potential multicollinearity among covariates in the fully adjusted models, we calculated the variance inflation factor (VIF) for each variable. All VIFs were below the conventional threshold of 5, indicating that multicollinearity did not significantly affect the stability or precision of the model estimates.

For outcomes at 30 days, adjusted odds ratios (aORs) with 95% CIs were calculated using multivariate logistic regressions. Interaction terms between age and frailty categories were then used to calculate relative odds ratios (rORs) and *P*-interaction values. Given the number of analyses performed, our interpretation of findings focused on the magnitude and precision of effect estimates (i.e. HRs/ORs and their 95% CIs) and the consistency of results across related endpoints, rather than relying solely on statistical significance thresholds. Statistical analysis was conducted using Stata 18.0 (StataCorp, College Station, TX, USA).

## Results

### Patient characteristics

Among the 931 133 patients included (see Supplementary data online, Figure S1), 156 617 (17%) were young (<55 years), 410 569 (44%) were middle-aged (55–74 years), and 363 947 (39%) were older (≥75 years). Older patients tended to be female, have a higher prevalence of co-morbidities and present with NSTEMI. Young patients had the highest median body mass index and were most likely to smoke. Young patients were more likely to receive invasive coronary angiography followed by revascularization with PCI. The greatest percentage of patients who underwent CABG surgery were middle-aged (Table 1). According to the SCARF index, in the overall cohort, 29% were classified as fit, 36% with mild frailty, 22% with moderate frailty, and 13% with severe frailty.

Table 2 compares demographic data across age groups of AMI patients stratified by frailty status. The SCARF index classified 3710 (2.4%) of patients under 55 years as severely frail, increasing to 34 259 (8.3%) in those aged 55–74 years and 79 114 (21.7%) in patients aged 75 years and older. Young and middle-aged severely frail patients had a higher body mass index than older severely frail patients and were more likely to be Asian, to smoke, and have a family history of coronary artery disease. Diabetes and peripheral vascular disease were also more prevalent in young and middle-aged patients with severe frailty compared with patients aged 75 years or older. In contrast, older severely frail patients were more likely to be female and have a higher prevalence of cognitive and mental health problems, chronic kidney disease, and cerebrovascular disease. In patients with severe frailty, malignancy was more prevalent in older patients (4.9%) compared with young (0.8%) and middle-aged (3.1%) individuals.

The age-specific distributions of each individual SCARF index health deficit are visualized in Supplementary data online, Figure S2. In younger patients, the most common deficits were diabetes, hypertension, HF, and prior ischaemic heart disease, indicating these were key contributors to frailty in this population. Clinical characteristics and in-hospital treatments according to age and frailty status are shown in Supplementary data online, Table S3. Notably, increasing frailty in young patients was associated with higher rates of CABG surgery, whereas the opposite trend was observed in older patients. Rates of PCI decreased with increasing frailty severity across all age groups.

**Figure 1:**
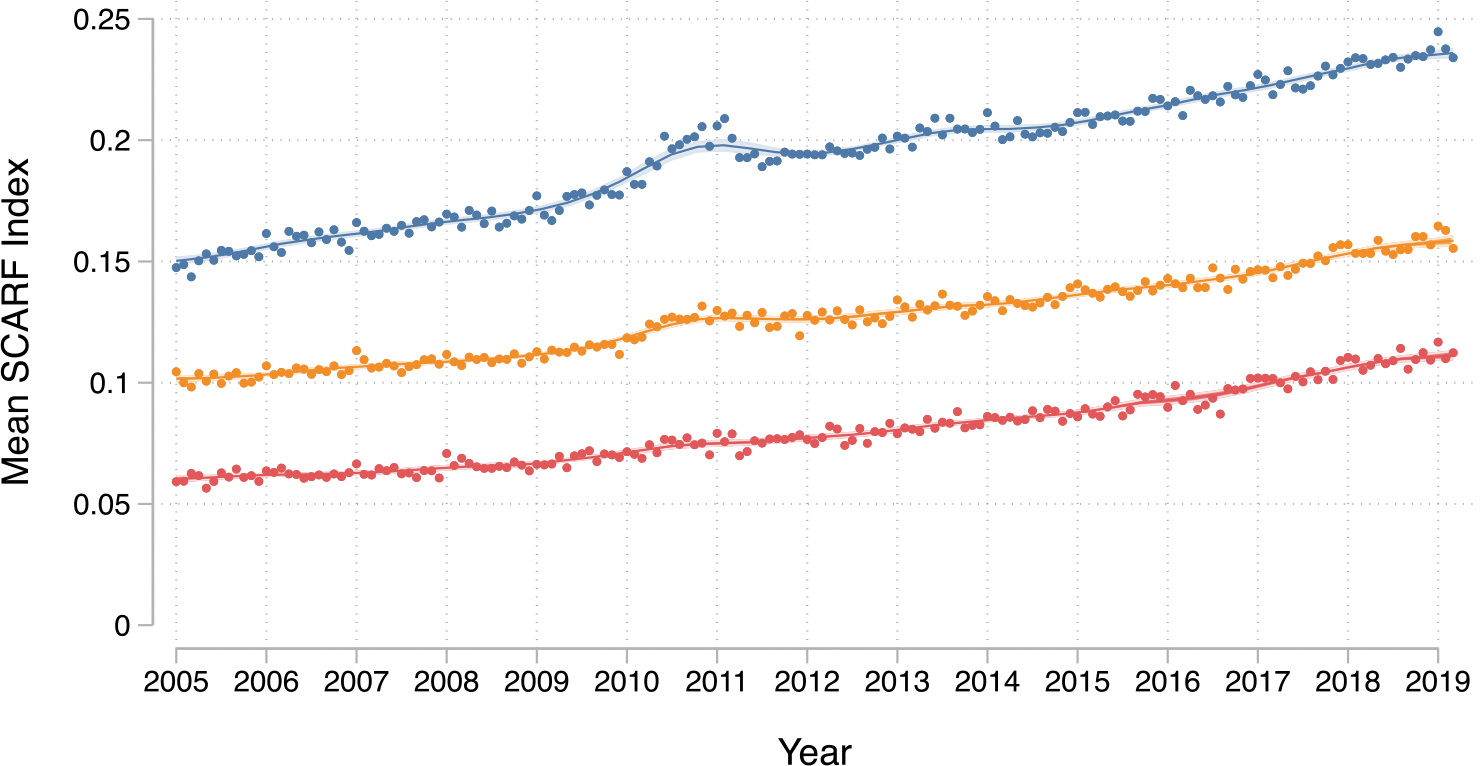
Trends in frailty over time (mean Secondary Care Administrative Records Frailty index by month) in patients with acute myocardial infarction stratified by age (years). Labels: Blue circle (top line), ≥75 years; orange circle (middle line), 55–74 years; red circle (bottom line), <55 years

Figure 1 shows the trends in mean frailty scores over time, stratified by age group. Frailty scores increased gradually across all age groups throughout the study period. Supplementary data online, Figure S3 displays the prevalence trends of the 31 individual health deficits comprising the SCARF index. The consistent increase observed across nearly all deficits suggests a genuine rise in levels of frailty within the AMI population, rather than an artefact of coding practices.

### European Society of Cardiology quality of care indicators

Supplementary data online, Figure S4 summarizes adherence to ESC quality indicators and overall OBQI scores, revealing disparities in the care of frailer AMI patients across all age groups. This included the uptake of timely invasive management, admission under cardiology care, prescription of secondary preventative therapies, and referrals for cardiac rehabilitation.

### Outcomes

#### Association of frailty with 1-year outcomes

**Figure 2:**
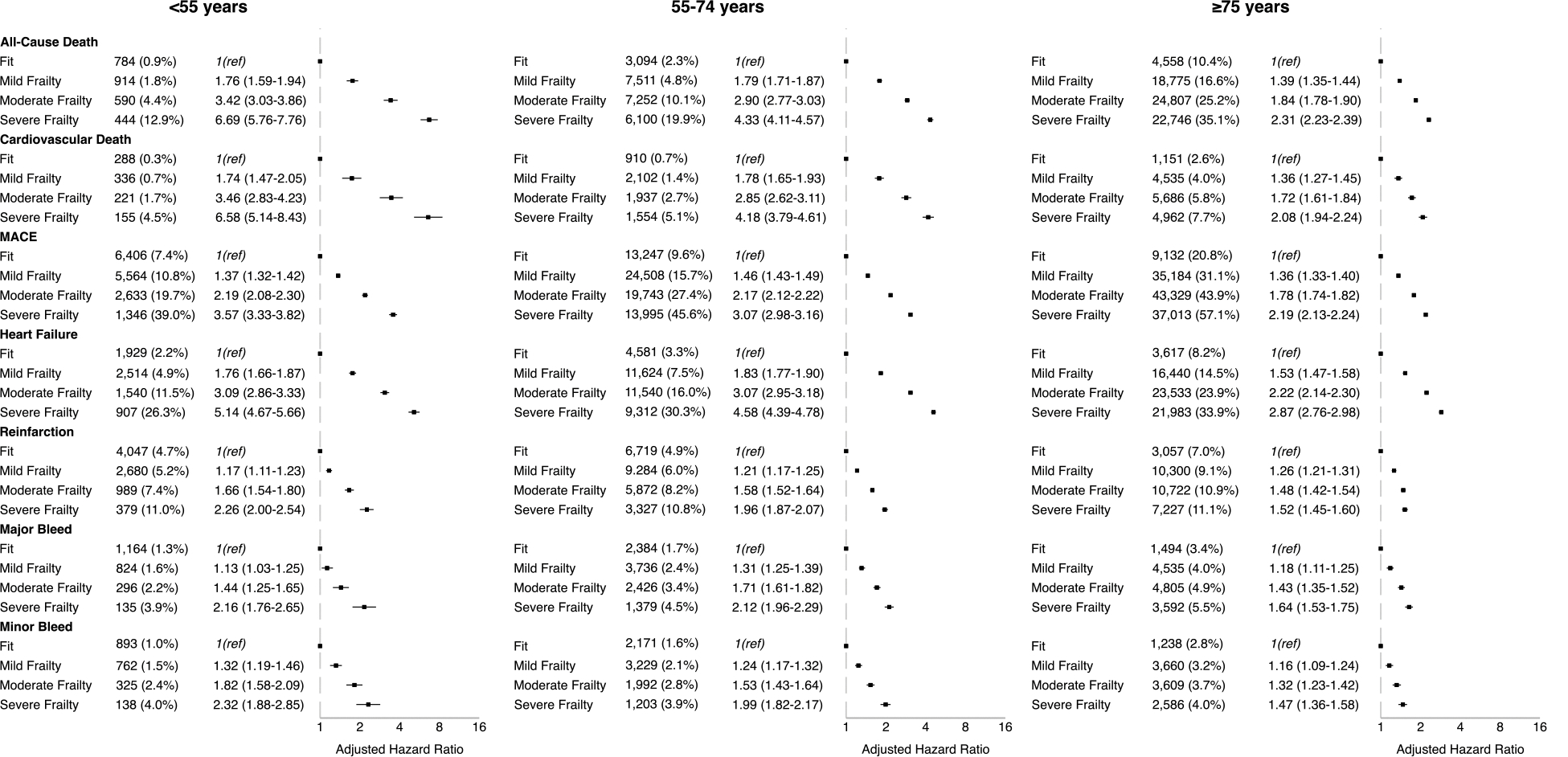
Absolute rates (%) and adjusted hazard ratios (with 95% confidence intervals) of outcomes at 1-year in patients with acute myocardial infarction stratified by age (years) and frailty status

Adjusted HRs for all-cause mortality at 1 year demonstrated a stepwise increase with increasing frailty severity across all age groups, when compared with fit patients (Figure 2). This was particularly evident in the young cohort, where aHRs for all-cause mortality ranged from 1.76 (95% CI 1.59–1.94) for mild frailty to 6.69 (95% CI 5.76–7.76) for severe frailty compared with young ‘fit’ patients. In older patients aged ≥75, aHRs ranged from 1.39 (95% CI 1.35–1.44) in mild frailty to 2.31 (95% CI 2.23–2.39) in severe frailty. Similar trends of increasing risk with rising levels of frailty were observed for cardiovascular mortality, MACE, HF, reinfarction, and bleeding events. Crude event rates were consistently lowest in young, fit patients.

#### Interaction between age and frailty

**Figure 3:**
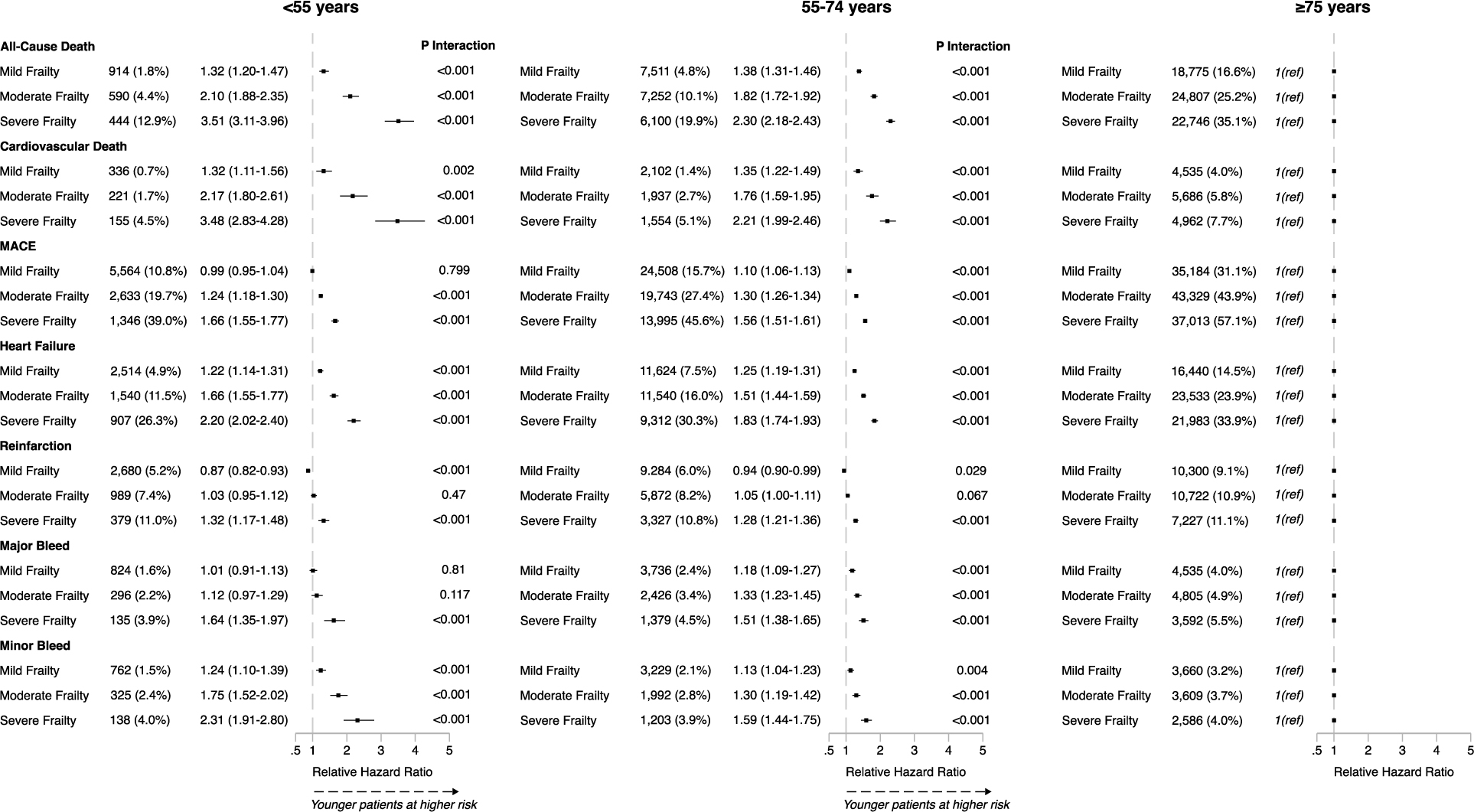
Relative hazard ratios (with 95% confidence intervals) of outcomes at 1 year in young and middle-aged frail patients compared with older adults (aged ≥75 years)

To explore the impact of frailty in younger patients with AMI, an interaction analysis was conducted between age and frailty categories, using older patients (≥75 years) as the comparator group. This demonstrated a significant interaction for all outcomes, indicating the association of frailty with all-cause mortality and secondary outcomes was stronger in younger patients compared with older patients with similar levels of frailty (Figure 3). In young patients, the rHR for all-cause mortality in mild frailty was 1.32 (95% CI 1.20–1.47, *P* < .001), increasing to 3.51 (95% CI 3.11–3.96, *P* < .001) for patients with severe frailty. Young patients with severe frailty also showed a significantly higher risk of cardiovascular and bleeding-related events, when compared with older severely frail adults. When age and the SCARF frailty score were analysed as continuous variables, a significant interaction was observed for all primary and secondary outcomes at 1 year and 30 days (all *P* ≤ .002; Supplementary data online, Table S4).

#### Years of life lost

Supplementary data online, Figure S5 summarizes the impact of frailty on mean YLL at 1 year following AMI. Young patients with severe frailty experienced the greatest YLL, reflecting the loss of greater potential life years due to premature mortality. Within the young cohort, YLL increased substantially with increasing frailty severity, ranging from 0.8 years for fit individuals to 6.1 years for severe frailty.

#### Sensitivity analysis

A sensitivity analysis of 30-day outcomes, including in-hospital events, demonstrated a higher odd of all-cause mortality with worsening frailty, mirroring the 1-year analysis (see Supplementary data online, Table S5). Compared with older patients, young patients with severe frailty had the highest relative odds of all-cause and cardiovascular death, HF, and major bleeding (see Supplementary data online, Table S6). Unadjusted event rates are summarized in Supplementary data online, Table S7.

## Discussion

This nationwide study of nearly one million patients hospitalized with AMI in England and Wales provides the first large-scale evidence of the profound impact of frailty on outcomes in younger individuals. Overall, we found that 1 in 10 patients with AMI present with severe frailty. Importantly, young severely frail patients with AMI experienced a nearly four-fold higher relative risk of all-cause mortality when compared with older patients (*Structured Graphical Abstract*). This striking disparity underscores the importance of incorporating robust frailty assessment into routine AMI care, even for younger patients. Future research should explore targeted interventions—such as comprehensive geriatric assessment, rehabilitation, and psychosocial support—to improve outcomes in this vulnerable group.^27^

The SCARF index classified only 29% of patients in this AMI cohort as ‘fit’, a figure potentially lower than anticipated in general clinical practice. This distribution likely reflects the inherent illness burden in AMI patients and the SCARF index’s sensitivity to common cardiovascular comorbidities (such as hypertension, diabetes, and HF), which are highly prevalent but may not be perceived as components of frailty in clinical practice. The stringent ‘fit’ definition (0–1 deficits) means even two deficits often result in a ‘mild frailty’ classification, a group likely encompassing many ‘pre-frail’ individuals with adequate physiological reserves but with a diminished capacity to recover from stressors.^28^ Critically, our study also identified that over a third of patients (35%) presented with moderate (22%) or severe (13%) frailty. This substantial burden of established frailty, where the prognostic implications are most pronounced, is consistent with the widely recognized prevalence of significant vulnerability in broader AMI populations and underscores the clinical importance of these higher-risk groups.

Our study reveals that the mechanisms underlying frailty and its adverse outcomes differ significantly by age, a distinction crucial for clinical interpretation. Our detailed analysis of deficit distribution demonstrates a distinct ‘frailty phenotype’ in younger patients, driven by an accelerated accumulation of cardiovascular and metabolic comorbidities. This contrasts with frailty in older patients, who more frequently exhibit traditional geriatric syndromes such as falls and arthritis, aligning more closely with the conventional concept of frailty. This finding challenges the perception of frailty as a solely an age-related syndrome of physical decline, suggesting it can manifest in the young as heightened vulnerability from chronic organ-system disease.^7^ The predominance of cardiovascular deficits in young frail AMI patients likely confers acute risk by directly compromising the stressed cardiovascular system,^29^ which may partly explain their markedly increased relative risk compared with the young ‘fit’ individuals. Furthermore, a high deficit burden at a young age may reflect a susceptibility to accelerated disease processes that are more difficult to treat or control,^30^ with our data suggesting links to obesity, Asian ethnicity, and smoking. Notably, malignancy was not a primary driver of this early-onset frailty, being less prevalent in young severely frail patients compared with their older counterparts. Overall, these findings characterize two distinct phenotypes of frailty: a cardiovascular-centric one that drives the high relative risk in the young and a multifactorial geriatric profile that drives the high absolute risk in the older population.

A key question arising from these distinct deficit profiles is whether the ‘young phenotype’ should be interpreted as frailty or simply as high-risk cardiovascular multimorbidity. We argue it represents a true state of frailty, grounded in the established cumulative deficit model. This model defines frailty not by the specific type of health problem, but by the quantity of accumulated deficits, which collectively erode physiological reserve and confer a state of heightened vulnerability to stressors.^5^ While the pathway to this vulnerable state in our young cohort is through an accumulation of cardiovascular and metabolic diseases, the outcome—a dramatically increased risk of mortality following a stressor (the AMI)—is the cardinal feature of a frail state. In this context, frailty can be viewed as a manifestation of accelerated biological ageing, where a younger individual’s health status and vulnerability to stressors mirror that of a much older person. Therefore, we interpret this ‘young phenotype’ not merely as a list of comorbidities, but as a distinct and clinically crucial form of frailty.

The interplay between age, baseline risk, and frailty significantly influences the interpretation of frailty’s prognostic impact, particularly when distinguishing between relative and absolute risk. As demonstrated by the crude event rates, because ‘fit’ young patients exhibit the lowest baseline event rates (0.7% in-hospital mortality), any slight increase in their absolute risk (7.0% in-hospital mortality in severely frail young patients) results in a far greater relative risk as their baseline risk is so low. The development of frailty within this younger demographic is therefore associated with markedly increased relative risk for adverse outcomes. This illustrates a key epidemiological principle of effect modification by baseline risk; whereby the low absolute risk in young, fit individuals statistically amplifies the relative impact of frailty. Consequently, the development of frailty in a younger person signifies a greater proportional deviation from their otherwise expected low-risk trajectory compared with older patients. This underscores that for young, severely frail patients, chronological age alone is an insufficient marker of their true underlying vulnerability and significantly higher risk profile.

These findings have significant clinical implications. The premature mortality observed in young frail patients, highlighted by our YLL analysis, underscores the need for better risk stratification beyond age alone.^31^ Yet, there remains an evidence gap in optimal frailty screening for younger individuals, as most tools were developed for older populations.^5,32^ This evidence gap extends to management; the observed disparities in adherence to key ESC quality indicators for frail patients likely contribute to their poorer outcomes.^33^ Deficits in acute care processes like timely invasive management, which are emphasized in recent ESC acute coronary syndrome guidelines^16^ for their impact on 30-day outcomes, along with lower adherence to established secondary prevention strategies at discharge, critical for 1-year prognosis, may partly explain the adverse events documented in this vulnerable group. Furthermore, the risk-benefit of invasive interventions also remains unclear, as major randomized controlled trials excluded younger frail patients, though observational data suggest potential benefits.^17,33,34^ Our findings highlight the importance of optimizing guideline-directed medical therapy for HF, a key driver of MACE in this cohort, and improving the uptake of cardiac rehabilitation, which is vital for addressing the multifaceted needs of frailer individuals.^35–38^

### Strengths and limitations

This study leverages the strengths of a large national dataset with linked outcomes. Here, we apply the SCARF index, which offers a more complete characterization of frailty domains when compared with older tools like the Hospital Frailty Risk Score (HFRS).^18^ While HFRS benefits from ease of calculation (using 109 ICD-10 codes)^39^ and has been widely validated,^40^ it can potentially overlook crucial functional deficits^41^ and has shown limitations in accurately predicting outcomes in critically ill populations.^42^ In contrast, the SCARF index encompasses a broader range of health deficits, consisting of 356 ICD-10 codes that directly relate to the domains of a comprehensive geriatric assessment, aligning strongly with the theoretical underpinnings of frailty.^5,18^ It has demonstrated robust predictive validity^18,19^ and is based on the Electronic Frailty Index, which is the most commonly used primary care FI, now widely implemented across general practices in England.^18,43^ Our study, using the SCARF index, has allowed for a more nuanced understanding of frailty’s impact in AMI, particularly among younger patients.

It is important to interpret our findings within the context of its limitations. As an epidemiological study based on administrative registries, our analyses can identify associations but cannot establish causal relationships between frailty and adverse outcomes. While we adjusted for a comprehensive set of demographic and clinical covariates available in the linked datasets, data from administrative registries are inherently prone to potential unmeasured or residual confounding factors that could influence the observed results. The MINAP and HES-APC also rely on self-reported in-hospital adverse events without external validation. However, fatal events were extracted from ONS, which records all deaths in England and Wales.

Additionally, frailty was assessed using a database-driven approach rather than clinical measures, such as the Clinical Frailty Scale or comprehensive geriatric assessment, which may have limited the granularity of frailty measurement in this study. For instance, clinical syndromes like falls may be under-recorded in administrative data unless they are severe enough to result in a specific hospital admission, a complication while an in-patient such as a fracture or are coded as part of another diagnosis. The SCARF index was initially developed and validated in patients with breast cancer.^18^ While this initial focus could be seen as a limitation regarding its direct applicability to an AMI population, many of the deficits included in SCARF (such as cardiovascular comorbidities, diabetes, renal disease, and cerebrovascular disease) are highly relevant to and prevalent in patients with AMI. While direct validation of SCARF across diverse cardiovascular cohorts is an ongoing area for research, its comprehensive nature and foundation on established frailty principles allow for meaningful risk stratification in our AMI population.

## Conclusion

Frailty is associated with adverse outcomes in patients with AMI, particularly in young patients with severe frailty. These findings underscore the importance of considering frailty in the risk stratification and care of AMI patients across all age groups. Future research should prioritize the development and evaluation of optimal frailty assessment tools and treatment strategies for young AMI patients.

## Supplementary Material

Supp Material 1

Supp Material 2

Supp Material 4

Supp Material 5

Supp Material 7

Supp Material 6

Supp Material 3

Supp Material 8

Supp Material 9

Supp Material 10

Supp Material 11

Supp Material 12

Supplementary data

Supplementary data are available at *European Heart Journal* online.

## Figures and Tables

**Table 1:** Characteristics of patients with acute myocardial infarction according to age group

	Age category (years)			
	<55	55–74	75+	Overall

N	156 617 (16.8%)	410 569 (44.1%)	363 947 (39.1%)	931 133 (100%)
Patient demographics				
SCARF frailty category				
Fit (FI < 0.05)	86 872 (55.5%)	139 838 (34.1%)	47 326 (13.0%)	274 036 (29.4%)
Mild (FI = 0.06–0.11)	52 221 (33.3%)	160 319 (39.0%)	124 649 (34.2%)	337 189 (36.2%)
Moderate (FI = 0.12–0.18)	13 814 (8.8%)	76 153 (18.5%)	112 858 (31.0%)	202 825 (21.8%)
Severe (FI > 0.19)	3710 (2.4%)	34 259 (8.3%)	79 114 (21.7%)	117 083 (12.6%)
Female	30 566 (19.5%)	113 979 (27.8%)	173 422 (47.7%)	317 967 (34.1%)
Ethnicity				
White	67 716 (86.9%)	189 491 (91.8%)	171 045 (94.8%)	428 252 (92.1%)
Black	1360 (1.7%)	2002 (1.0%)	1631 (0.9%)	4993 (1.1%)
Asian	8886 (11.4%)	15 015 (7.3%)	7782 (4.3%)	31 683 (6.8%)
BMI, median (IQR)	28.4 (32.1–25.3)	27.6 (24.7–31.1)	25.5 (22.7–28.7)	27.0 (24.0–30.5)
Current smoker	85 363 (57.6%)	118 393 (30.8%)	28 941 (8.8%)	232 697 (27.0%)
Diabetes	26 331 (16.8%)	102 679 (25.0%)	93 057 (25.6%)	222 067 (23.8%)
Hypertension	61 023 (39.0%)	231 464 (56.4%)	240 009 (65.9%)	532 496 (57.2%)
Hypercholesterolaemia	42 748 (30.4%)	139 164 (37.5%)	101 295 (30.7%)	283 207 (33.7%)
Peripheral vascular disease	5601 (3.6%)	32 705 (8.0%)	36 354 (10.0%)	74 660 (8.0%)
Chronic kidney disease	4249 (2.7%)	27 308 (6.7%)	64 903 (17.8%)	87 889 (9.4%)
History of respiratory disease	21 493 (13.7%)	91 174 (22.2%)	110 836 (30.5%)	223 503 (24.0%)
Heart failure	22 511 (14.4%)	81 801 (19.9%)	117 143 (32.2%)	221 455 (23.8%)
Stroke/TIA	4126 (2.6%)	29 411 (7.2%)	54 352 (14.9%)	87 889 (9.4%)
Cognitive and mental health problems	4602 (2.9%)	12 341 (3.0%)	39 580 (10.9%)	56 523 (6.1%)
Family history of CAD	60 090 (47.9%)	110 518 (35.3%)	42 629 (16.8%)	213 237 (30.8%)
History of ischaemic heart disease	30 980 (19.8%)	138 756 (33.8%)	169 869 (46.7%)	339 605 (36.5%)
Any malignancy	783 (0.5%)	9596 (2.3%)	17 033 (4.7%)	27 412 (2.9%)
Metastatic solid cancer	248 (0.2%)	2451 (0.6%)	3214 (0.9%)	5913 (0.6%)
Charlson comorbidity index				
CCI = 0–1	126 332 (80.7%)	262 737 (64.0%)	163 556 (44.9%)	552 625 (59.3%)
CCI = 2–3	25 286 (16.1%)	107 773 (26.2%)	130 340 (35.8%)	263 399 (28.3%)
CCI > 3	4999 (3.2%)	40 059 (9.8%)	70 051 (19.2%)	115 109 (12.4%)
Clinical characteristics				
Left ventricular ejection fraction (echocardiogram)				
Good (≥50%)	46 015 (63.4%)	106 847 (57.2%)	70 921 (47.5%)	223 783 (54.8%)
Moderate (30%-49%)	21 802 (30.1%)	61 033 (32.7%)	54 243 (36.3%)	137 078 (33.5%)
Poor(<30%)	4707 (6.5%)	18 813 (10.1%)	24 257 (16.2%)	47 777 (11.7%)
Systolic blood pressure, median (IQR)	136 (120–154)	138 (120–157)	137 (118–157)	137 (120–156)
Heart rate, median (IQR)	77 (66–90)	76 (65–90)	80 (67–95)	78 (66–92)
Creatinine, μmol/L, median (IQR)	80 (69–93)	86 (72–102)	99 (79–130)	88 (74–110)
Killip class				
Killip class I	62 498 (78.2%)	137 318 (58.6%)	79 648 (32.6%)	279 464 (50.1%)
Killip class II	13 540 (16.9%)	79 914 (34.1%)	139 764 (57.3%)	233 218 (41.8%)
Killip class III	1044 (1.3%)	6772 (2.9%)	12 821 (5.3%)	20 637 (3.7%)
Killip class IV	2829 (3.5%)	10 194 (4.4%)	11 719 (4.8%)	24 742 (4.4%)
STEMI	76 644 (48.9%)	161 644 (39.4%)	95 182 (26.2%)	333 470 (35.8%)
NSTEMI	79 973 (51.1%)	248 925 (60.6%)	268 765 (73.8%)	597 663 (64.2%)
Cardiac arrest	9485 (6.3%)	24 982 (6.3%)	23 825 (6.8%)	58 292 (6.5%)
Medications prescribed				
Dual antiplatelets	128 393 (93.3%)	322 427 (90.2%)	248 523 (79.8%)	699 343 (86.7%)
Fondaparinux or LMWH	87 712 (67.9%)	248 206 (72.4%)	235 678 (75.7%)	571 596 (73.0%)
Unfractionated heparin	42 206 (33.4%)	95 846 (28.8%)	51 058 (16.9%)	189 110 (24.8%)
Warfarin	2483 (2.0%)	14 679 (4.4%)	24 377 (8.0%)	41 539 (5.5%)
Glycoprotein IIb/IIIa inhibitors	16 938 (13.2%)	33 086 (9.7%)	13 173 (4.3%)	63 197 (8.1%)
ACE inhibitor or ARB	118 520 (89.1%)	305 503 (87.1%)	233 097 (75.3%)	657 120 (82.8%)
Beta-blocker	126 535 (90.1%)	315 997 (86.0%)	249 276 (77.6%)	691 808 (83.5%)
High-dose statin	129 977 (96.3%)	344 108 (95.6%)	279 575 (88.5%)	753 660 (93.0%)
Mineralocorticoid receptor antagonist	7432 (8.5%)	23 771 (10.8%)	23 088 (12.1%)	54 291 (10.9%)
Interventional management				
Invasive coronary angiogram	98 457 (75.8%)	227 073 (68.7%)	109 552 (42.0%)	435 082 (60.3%)
PCI	87 450 (67.3%)	189 468 (57.3%)	86 765 (33.2%)	363 683 (50.4%)
CABG surgery	2661 (2.1%)	11 382 (3.5%)	5036 (2.0%)	19 079 (2.7%)

**Table 2:** Demographic data in patients with acute myocardial infarction stratified by age and frailty groups

	Age group (years)											
	<55 years				55–74 years				≥75 years			
	Fit	Mild frailty	Moderate frailty	Severe frailty	Fit	Mild frailty	Moderate frailty	Severe frailty	Fit	Mild frailty	Moderate frailty	Severe frailty

*N*	86 872 (55.5%)	52 221 (33.3%)	13 814(8.8%)	3710(2.4%)	139 838 (34.1%)	160 319 (39.1%)	76153 (18.6%)	34259 (8.3%)	47 326 (13.0%)	124649 (34.3%)	112858 (31.0%)	79114 (21.7%)
Female	14 336 (16.5%)	11 094 (21.2%)	3943 (28.5%)	1193 (32.2%)	33 148 (23.7%)	45 033 (28.1%)	24 013 (31.5%)	11 785 (34.4%)	20119 (42.5%)	58 102 (46.6%)	55 114 (48.8%)	40 087 (50.7%)
Ethnicity												
White	34436 (88.7%)	23 813 (85.5%)	7374 (84.7%)	2093 (81.5%)	58 435 (95.5%)	73 422 (91.7%)	38 384 (89.0%)	19 250 (86.9%)	17680 (97.1%)	52193 (95.6%)	54 925 (94.6%)	46 247 (93.2%)
Black	565 (1.5%)	550 (2.0%)	169 (1.9%)	76 (3.0%)	374 (0.6%)	736 (0.9%)	530 (1.2%)	362 (1.6%)	99 (0.5%)	381 (0.7%)	507 (0.9%)	644 (1.3%)
Asian	3827 (9.9%)	3500 (12.6%)	1161 (13.3%)	398 (15.5%)	2407 (3.9%)	5882 (7.3%)	4190 (9.7%)	2536 (11.5%)	429 (2.4%)	2032 (3.7%)	2608 (4.5%)	2713 (5.5%)
BMI, median (IQR)	27.7(24.9–31.0)	29.1 (25.7–33.0)	30.1 (26.2–34.5)	30.4 (25.6–35.9)	26.8 (24.3–29.7)	27.7 (24.7–31.1)	28.6 (25.1–32.6)	29.3 (25.4–33.9)	25.1 (22.7–27.7)	25.4 (22.8–28.4)	25.6 (22.7–28.9)	25.6 (22.5–29.3)
Current smoker	50425 (61.0%)	26 804 (54.4%)	6638 (51.3%)	1496 (43.4%)	46 894 (35.6%)	44 656 (29.7%)	19 347 (27.2%)	7496 (23.7%)	4977 (11.7%)	10468 (9.3%)	8363 (8.2%)	5133 (7.3%)
Diabetes	3215 (3.7%)	13 770 (26.4%)	6816 (49.3%)	2530 (68.2%)	4590 (3.3%)	38 606 (24.1%)	36 414 (47.8%)	23 069 (67.3%)	1280 (2.7%)	20 562 (16.5%)	34 744 (30.8%)	36 471 (46.1%)
Hypertension	15 508 (17.9%)	32 008 (61.3%)	10440 (75.6%)	3067 (82.7%)	34 836 (24.9%)	106 644 (66.5%)	60 487 (79.4%)	29497 (86.1%)	13 315 (28.1%)	78 010(62.6%)	83 977 (74.4%)	64707 (81.8%)
Hypercholesterolaemia	15 935 (20.5%)	19 153 (40.7%)	5991 (47.7%)	1669 (48.8%)	30 377 (24.4%)	60 698 (41.9%)	32 858 (47.2%)	15 231 (48.1%)	7421 (18.0%)	34 009 (30.3%)	34 468 (33.3%)	25 397 (34.7%)
Peripheral vascular disease	569 (0.7%)	2319 (4.4%)	1645 (11.9%)	1068 (28.8%)	1223 (0.9%)	9082 (5.7%)	11 817 (15.5%)	10583 (30.9%)	400 (0.8%)	5914 (4.7%)	12 792 (11.3%)	17248 (21.8%)
Chronic kidney disease	159 (0.2%)	1209 (2.3%)	1481 (10.7%)	1400 (37.7%)	340 (0.2%)	4740 (3.0%)	9535 (12.5%)	12 693 (37.1%)	347 (0.7%)	7971 (6.4%)	22 646 (20.1%)	33 939 (42.9%)
History of respiratory disease	4645 (5.3%)	10 065 (19.3%)	4883 (35.3%)	1900 (51.2%)	8770 (6.3%)	34 315 (21.4%)	29119 (38.2%)	18 970 (55.4%)	3267 (6.9%)	26 638 (21.4%)	40 018 (35.5%)	40913 (51.7%)
Heart failure	3888 (4.5%)	10 535 (20.2%)	5696 (41.2%)	2392 (64.5%)	5320 (3.8%)	26 279 (16.4%)	28 457 (37.4%)	21 745 (63.5%)	1664 (3.5%)	21 133 (17.0%)	44 299 (39.3%)	50 047 (63.3%)
Stroke/TIA	337 (0.4%)	1620 (3.1%)	1394 (10.1%)	775 (20.9%)	907 (0.6%)	8155 (5.1%)	10925 (14.3%)	9424 (27.5%)	693 (1.5%)	10098 (8.1%)	19 867 (17.6%)	23 694 (29.9%)
Cognitive and mental health problems	802 (0.9%)	2091 (4.0%)	1223 (8.9%)	486 (13.1%)	774 (0.6%)	3831 (2.4%)	3971 (5.2%)	3765 (11.0%)	696 (1.5%)	7289 (5.8%)	13 322 (11.8%)	18 273 (23.1%)
Family history of CAD	34078 (48.6%)	20 227 (48.6%)	4733 (43.3%)	1052 (36.6%)	41 692 (38.4%)	44121 (36.1%)	18113 (31.8%)	6592 (26.2%)	6363 (19.2%)	16 083 (18.4%)	12 760 (16.2%)	7423 (13.5%)
History of ischaemic heart disease	5100 (5.9%)	16 872 (32.3%)	6810 (49.3%)	2198 (59.2%)	10 250 (7.3%)	60 940 (38.0%)	43 973 (57.7%)	23 593 (68.9%)	5143 (10.9%)	50 585 (40.6%)	62 877 (55.7%)	51 264 (64.8%)
Any malignancy	390 (0.4%)	266 (0.5%)	97 (0.7%)	30 (0.8%)	2523 (1.8%)	3804 (2.4%)	2211 (2.9%)	1058 (3.1%)	1865 (3.9%)	5647 (4.5%)	5631 (5.0%)	3890 (4.9%)
Metastatic solid cancer	134 (0.2%)	81 (0.2%)	25 (0.2%)	8 (0.2%)	622 (0.4%)	1025 (0.6%)	599 (0.8%)	205 (0.6%)	404 (0.9%)	1165 (0.9%)	1048 (0.9%)	597 (0.8%)
Charlson comorbidity index												
CCI = 0–1	84474 (97.2%)	36 914 (70.7%)	4539 (32.9%)	405 (10.9%)	133 718 (95.6%)	106 866 (66.7%)	19 636 (25.8%)	2517(7.3%)	44 209 (93.4%)	79 812(64.0%)	31 593 (28.0%)	7942 (10.0%)
CCI = 2–3	2213 (2.5%)	14 748 (28.2%)	7073 (51.2%)	1252 (33.7%)	5381 (3.8%)	49 944 (31.2%)	41 166 (54.1%)	11 282 (32.9%)	2645 (5.6%)	40 534 (32.5%)	58 521 (51.9%)	28 640 (36.2%)
CCI >3	185 (0.2%)	559 (1.1%)	2202 (15.9%)	2053 (55.3%)	739 (0.5%)	3509 (2.2%)	15 351 (20.2%)	20460 (59.7%)	472 (1.0%)	4303 (3.5%)	22 744 (20.2%)	42532 (53.8%)

FI, frailty index; IQR, interquartile range; BMI, body mass index; TIA, transient ischaemic attack; CAD, coronary artery disease.

## Data Availability

The authors do not have authorization to share the data used for this study. Data can be accessed through contacting the National Institute for Cardiovascular Outcomes Research (NICOR) and National Health Service (NHS) Digital upon approval.

## References

[1] Gulati R, Behfar A, Narula J, Kanwar A, Lerman A, Cooper L, Acute myocardial infarction in young individuals. Mayo Clin Proc 2020;95:136–56. 10.1016/j.mayocp.2019.05.001 31902409

[2] Damluji AA, Huang J, Bandeen-Roche K, Forman DE, Gerstenblith G, Moscucci M, Frailty among older adults with acute myocardial infarction and outcomes from percutaneous coronary interventions. J Am Heart Assoc 2019;8:e013686. 10.1161/JAHA.119.013686 31475601 PMC6755849

[3] Kwok CS, Lundberg G, Al-Faleh H, Sirker A, Van Spall HGC, Michos ED, Relation of frailty to outcomes in patients with acute coronary syndromes. Am J Cardiol 2019;124:1002–11. 10.1016/j.amjcard.2019.07.003 31421814

[4] Bebb O, Smith FGD, Clegg A, Hall M, Gale CP. Frailty and acute coronary syndrome: a structured literature review. Eur Heart J Acute Cardiovasc Care 2017;7:166–75. 10.1177/2048872617700873 29064267 PMC7614831

[5] Mitnitski AB, Mogilner AJ, Rockwood K. Accumulation of deficits as a proxy measure of aging. ScientificWorldJournal 2001;1:321027. 10.1100/tsw.2001.58 PMC6084020 12806071

[6] Blodgett JM, Rockwood K, Theou O. Changes in the severity and lethality of age-related health deficit accumulation in the USA between 1999 and 2018: a population-based cohort study. Lancet Healthy Longev 2021;2:e96–104. 10.1016/S2666-7568(20)30059-3 36098163

[7] Hanlon P, Nicholl BI, Jani BD, Lee D, McQueenie R, Mair FS. Frailty and pre-frailty in middle-aged and older adults and its association with multimorbidity and mortality: a prospective analysis of 493 737 UK Biobank participants. Lancet Public Health 2018;3:e323–32. 10.1016/S2468-2667(18)30091-4 29908859 PMC6028743

[8] Fan J, Yu C, Guo Y, Bian Z, Sun Z, Yang L, Frailty index and all-cause and cause-specific mortality in Chinese adults: a prospective cohort study. Lancet Public Health 2020;5:e650–60. 10.1016/S2468-2667(20)30113-4 33271078 PMC7708389

[9] Damluji AA, Forman DE, Wang TY, Chikwe J, Kunadian V, Rich MW, Management of acute coronary syndrome in the older adult population: a scientific statement from the American Heart Association. Circulation 2023;147:e32–62. 10.1161/CIR.0000000000001112 36503287 PMC10312228

[10] Moledina SM, Rashid M, Nolan J, Nakao K, Sun LY, Velagapudi P, Addressing disparities of care in non-ST-segment elevation myocardial infarction patients without standard modifiable risk factors: insights from a nationwide cohort study. Eur J Prev Cardiol 2022;29:1084–92. 10.1093/eurjpc/zwab200 34897399

[11] Rashid M, Abramov D, Naseer MU, Van Spall HGC, Ahmed FZ, Lawson C, 15-Year trends, predictors, and outcomes of heart failure hospitalization complicating first acute myocardial infarction in the modern percutaneous coronary intervention era. Eur Heart J Open 2025;5:oeaf013–92. 10.1093/ehjopen/oeaf013 40078653 PMC11896973

[12] NHS-Health-Research-Authority. Research Ethics Service and Research Ethics Committees 2024. https://www.hra.nhs.uk/about-us/committees-and-services/res-and-recs/ (February 2025, date last accessed).

[13] NHS-Health-Research-Authority. Confidentiality Advisory Group 2024. https://www.hra.nhs.uk/about-us/committees-and-services/confidentiality-advisory-group/ (February 2025, date last accessed).

[14] Von Elm E, Altman DG, Egger M, Pocock SJ, Gøtzsche PC, Vandenbroucke JP. The Strengthening the Reporting of Observational Studies in Epidemiology (STROBE) statement: guidelines for reporting observational studies. Lancet 2007;370:1453–7. 10.1016/S0140-6736(07)61602-X 18064739

[15] Kotecha D, Asselbergs FW, Achenbach S, Anker SD, Atar D, Baigent C, CODE-EHR best practice framework for the use of structured electronic healthcare records in clinical research. BMJ 2022;378:e069048. 10.1136/bmj-2021-069048 36562446 PMC9403753

[16] Byrne RA, Rossello X, Coughlan JJ, Barbato E, Berry C, Chieffo A, 2023 ESC Guidelines for the management of acute coronary syndromes. Eur Heart J 2023;44:3720–826. 10.1093/eurheartj/ehad191 37622654

[17] Tromp J, Paniagua SMA, Lau ES, Allen NB, Blaha MJ, Gansevoort RT, Age dependent associations of risk factors with heart failure: pooled population based cohort study. BMJ 2021;372:n461. 10.1136/bmj.n461 33758001 PMC7986583

[18] Jauhari Y, Gannon MR, Dodwell D, Horgan K, Clements K, Medina J, Construction of the Secondary Care Administrative Records Frailty (SCARF) index and validation on older women with operable invasive breast cancer in England and Wales: a cohort study. BMJ Open 2020;10:e035395. 10.1136/bmjopen-2019-035395 PMC7223146 32376755

[19] Birch R, Taylor J, Rahman T, Audisio R, Pilleron S, Quirke P, A comparison of frailty measures in population-based data for patients with colorectal cancer. Age Ageing 2024;53:afae105. 10.1093/ageing/afae105 38783754 PMC11116828

[20] Charlson ME, Pompei P, Ales KL, MacKenzie CR. A new method of classifying prognostic comorbidity in longitudinal studies: development and validation. J Chronic Dis 1987;40:373–83. 10.1016/0021-9681(87)90171-8 3558716

[21] Schiele F, Aktaa S, Rossello X, Ahrens I, Claeys MJ, Collet J-P, 2020 update of the quality indicators for acute myocardial infarction: a position paper of the Association for Acute Cardiovascular Care: the study group for quality indicators from the ACVC and the NSTE-ACS guideline group. Eur Heart J Acute Cardiovasc Care 2021;10:224–33. 10.1093/ehjacc/zuaa037 33550362

[22] Lindahl B, Baron T, Erlinge D, Hadziosmanovic N, Nordenskjöld A, Gard A, Medical therapy for secondary prevention and long-term outcome in patients with myocardial infarction with nonobstructive coronary artery disease. Circulation 2017;135:1481–9. 10.1161/CIRCULATIONAHA.116.026336 28179398

[23] Antman E, Bassand J-P, Klein W, Ohman M, Lopez Sendon JL, Rydén L, Myocardial infarction redefined—a consensus document of The Joint European Society of Cardiology/American College of Cardiology committee for the redefinition of myocardial infarction. J Am Coll Cardiol 2000;36:959–69. 10.1016/S0735-1097(00)00804-4 10987628

[24] Office-for-National-Statistics. National life tables—life expectancy in the UK: 2020 to 2022. 2024. https://www.ons.gov.uk/peoplepopulationandcommunity/birthsdeathsandmarriages/lifeexpectancies/bulletins/nationallifetablesunitedkingdom/2020to2022 (January 2025, date last accessed).

[25] Rashid M, Rushton CA, Kwok CS, Kinnaird T, Kontopantelis E, Olier I, Impact of access site practice on clinical outcomes in patients undergoing percutaneous coronary intervention following thrombolysis for ST-segment elevation myocardial infarction in the United Kingdom. JACC Cardiovasc Interv 2017;10:2258–65. 10.1016/j.jcin.2017.07.049 29169494

[26] Rubin DB. Multiple Imputation for Nonresponse in Surveys. New York: John Wiley & Sons, 2004

[27] Fountotos R, Lauck S, Piazza N, Martucci G, Arora R, Asgar A, Protein and exercise to reverse frailty in older men and women undergoing transcatheter aortic valve replacement: design of the PERFORM-TAVR trial. Can J Cardiol 2024;40:267–74. 10.1016/j.cjca.2023.11.037 38052302

[28] Rasiah J, Cummings GG, Gruneir A, Oelke ND, Estabrooks C, Holroyd-Leduc J. Prefrailty in older adults: a concept analysis. Int J Nurs Stud 2020;108:103618. 10.1016/j.ijnurstu.2020.103618 32450406

[29] James K, Jamil Y, Kumar M, Kwak MJ, Nanna MG, Qazi S, Frailty and cardiovascular health. J Am Heart Assoc 2024;13:e031736. 10.1161/JAHA.123.031736 39056350 PMC11964060

[30] Ahmed W, Muhammad T, Akhtar SN, Ali WK. Association of early and late onset of chronic diseases with physical frailty among older Indian adults: study based on a population survey. BMC Public Health 2025;25:688. 10.1186/s12889-025-21706-7 39972305 PMC11841361

[31] Damluji AA, Nanna MG, Rymer J, Kochar A, Lowenstern A, Baron SJ, Chronological vs biological age in interventional cardiology: a comprehensive approach to care for older adults: JACC Family Series. JACC Cardiovasc Interv 2024;17:961–78. 10.1016/j.jcin.2024.01.284 38597844 PMC11097960

[32] Spiers GF, Kunonga TP, Hall A, Beyer F, Boulton E, Parker S, Measuring frailty in younger populations: a rapid review of evidence. BMJ Open 2021;11:e047051. 10.1136/bmjopen-2020-047051 PMC7986767 33753447

[33] Sanchis J, Bueno H, Miñana G, Guerrero C, Martí D, Martínez-Sellés M, Effect of routine invasive vs conservative strategy in older adults with frailty and non–st-segment elevation acute myocardial infarction: a randomized clinical trial. JAMA Intern Med 2023;183:407–15. 10.1001/jamainternmed.2023.0047 36877502 PMC9989957

[34] Roman M, Miksza J, Lai FY-L, Sze S, Poppe K, Doughty R, Revascularization in frail patients with acute coronary syndromes: a retrospective longitudinal study. Eur Heart J 2024;46:53547. 10.1093/eurheartj/ehae755 PMC11804245 39548842

[35] McMurray JJV, Solomon SD, Inzucchi SE, Køber L, Kosiborod MN, Martinez FA, Dapagliflozin in patients with heart failure and reduced ejection fraction. N Engl J Med 2019;381:1995–2008. 10.1056/NEJMoa1911303 31535829

[36] Khan MS, Segar MW, Usman MS, Singh S, Greene SJ, Fonarow GC, Frailty, guideline-directed medical therapy, and outcomes in HFrEF: from the GUIDE-IT trial. JACC Heart Fail 2022;10:266–75. 10.1016/j.jchf.2021.12.004 35361446 PMC10539014

[37] Forman DE, Maurer MS, Boyd C, Brindis R, Salive ME, Horne FM, Multimorbidity in older adults with cardiovascular disease. J Am Coll Cardiol 2018;71:2149–61. 10.1016/j.jacc.2018.03.022 29747836 PMC6028235

[38] Lutz AH, Delligatti A, Allsup K, Afilalo J, Forman DE. Cardiac rehabilitation is associated with improved physical function in frail older adults with cardiovascular disease. J Cardiopulm Rehabil Prev 2020;40:310–8. 10.1097/HCR.0000000000000537 32804797

[39] Gilbert T, Neuburger J, Kraindler J, Keeble E, Smith P, Ariti C, Development and validation of a Hospital Frailty Risk Score focusing on older people in acute care settings using electronic hospital records: an observational study. Lancet 2018;391:1775–82. 10.1016/S0140-6736(18)30668-8 29706364 PMC5946808

[40] Street A, Maynou L, Gilbert T, Stone T, Mason S, Conroy S. The use of linked routine data to optimise calculation of the Hospital Frailty Risk Score on the basis of previous hospital admissions: a retrospective observational cohort study. Lancet Healthy Longev 2021;2:e154–62. 10.1016/S2666-7568(21)00004-0 33733245 PMC7934406

[41] Szakmany T, Hollinghurst J, Pugh R, Akbari A, Griffiths R, Bailey R, Frailty assessed by administrative tools and mortality in patients with pneumonia admitted to the hospital and ICU in Wales. Sci Rep 2021;11:13407. 10.1038/s41598-021-92874-w 34183745 PMC8239046

[42] Sy E, Kassir S, Mailman JF, Sy SL. External validation of the hospital frailty risk score among older adults receiving mechanical ventilation. Sci Rep 2022;12:14621. 10.1038/s41598-022-18970-7 36028750 PMC9418158

[43] Clegg A, Bates C, Young J, Ryan R, Nichols L, Ann TE, Development and validation of an electronic frailty index using routine primary care electronic health record data. Age Ageing 2016;45:353–60. 10.1093/ageing/afw039 26944937 PMC4846793

