# Supplementary material for: Frailty and long-term outcomes in younger patients with acute myocardial infarction
Source: Eur Heart J. 2025 Nov 25;47(21):2686–96. doi: 10.1093/eurheartj/ehaf876 (PMC12766437; doi:10.1093/eurheartj/ehaf876)
Supplement: ehaf876_Supplementary_Data [file ehaf876_supplementary_data.zip › Supplementary Table 1.docx]

**Supplementary Table 1** Variables used to construct SCARF Index and CCI

| ***Secondary Care Administrative Records Frailty Index*** | |
| --- | --- |
| Activity Limitation | R26 S78 S88 Y83 G11 G81 G82 G83 M62 T136 Z993 |
| Neurodegenerative Disorders | G20 G21 G22 G23 G25 G26 G32 G35 R25 G122 |
| Requirement for Care | R40 Z50 Z74 Z755 Z998 Z999 |
| Social Vulnerability | F10 Y06 Z59 Z60 Z63 Z73 R460 R468 |
| Hearing Impairment | H90 H91 H833 Z453 Z461 Z974 |
| Visual Impairment | H25 H28 H35 H40 H43 H53 H54 |
| Falls | S00 S01 W00 W01 W04 W05 W06 W07 W08 W10 W18 W19 R296 |
| Skin Ulcer | I83 I98 L03 L08 L89 L97 L984 |
| Incontinence | N31 R15 R32 N393 N394 T835 Z466 |
| Nutritional Problems | E41 E43 E44 E46 E53 E55 E66 E83 E87 R53 R63 R64 X53 R628 |
| Cognitive and Mental Health Problems | F00 F01 F02 F03 F04 F05 G30 G31 R41 R54 F2 F3 F41 R44 R45 F067 |
| Anaemia | D50 D51 D52 D53 D63 D64 |
| Arthritis | M05 M06 M07 M09 M10 M11 M12 M13 M15 M16 M17 M18 M19 M32 M34 M35 M36 M315 |
| Cardiac Arrhythmias | I44 I48 I49 Z450 Z950 |
| Cerebrovascular Disease | G45 G46 I60 I61 I62 I63 I64 I65 I66 I67 I68 I69  *MINAP variable: previous cerebrovascular disease* |
| Chronic Kidney Disease (CKD) | I12 I13 N01 N03 N05 N07 N08 N18 N19 N25 Z49 I770 Z940 Z992  *MINAP variable: chronic kidney disease* |
| Diabetes | E109 E119 E129 E139 E149  *MINAP variable: diabetes* |
| Diabetic Complications | E100 E101 E102 E103 E104 E105 E106 E107 E108 E110 E111 E112 E113 E114 E115 E116 E117 E118 E120 E121 E122 E123 E124 E125 E126 E127 E128 E130 E131 E132 E133 E134 E135 E136 E137 E138 E140 E141 E142 E143 E144 E145 E146 E147 E148 G590 G632 H360 M142 M146 N083 |
| Heart Failure | I11 I13 I27 I42 I43 I50 I51 I260  *MINAP variable: history of heart failure* |
| Heart Valve Disease | I05 I06 I07 I08 I34 I35 I37 I390 I391 I392 I393 I394 Z952 Z953 Z954 |
| Hypertension | I10 I11 I12 I13 H350  *MINAP variable: hypertension* |
| Hypotension | I95 R55 R42 E86  *MINAP variables: systolic blood pressure or Killip class IV* |
| Ischaemic Heart Disease (IHD) | I252  *MINAP variables: previous MI, previous PCI, previous CABG, previous angina* |
| Foot Problems | L60 S90 S91 S92 S93 S94 S96 S97 S99 Q66 B353 G575 G576 M201 M202 M203 M204 M205 M206 M213 M214 M215 M216 M722 M766 M773 M775 |
| Fragility Fracture | S22 S32 S33 S42 S43 S62 S72 S73 M484 |
| Osteoporosis | M80 M81 M82 |
| Peptic Ulcer Disease | K21 K25 K26 K27 K29 R12 |
| Peripheral Vascular Disease | I65 I70 I71 I72 I73 R02 I771 K551 K558 K559 Z958 Z959  *MINAP variable: history of peripheral vascular disease* |
| Respiratory Disease | J45 J46 J40 J41 J42 J43 J44 J47 J60 J61 J62 J63 J64 J65 J70 J13 J14 J15 J16 J18 J22 J90 R06 J684 J961 J980  *MINAP variable: history of asthma or COPD* |
| Thyroid Disease | E03 E04 E05 E06 E079 |
| Urinary System Disease | N30 N34 R31 R33 N390 N398 N399 T835 |
| ***Charlson Co-morbidity Index*** | |
| Myocardial Infarction | I254  *MINAP variable: previous MI* |
| Congestive Heart Failure | I50 I43 I09 I099 I110 I130 I132 I255  *MINAP variable: history of heart failure* |
| Peripheral Vascular Disease | I70 I71 I73 I77 I79 K55 Z95  *MINAP variable: history of peripheral vascular disease* |
| Cerebrovascular Disease | G45 G46 I60 I61 I62 I63 I64 I65 I66 I67 I68 I69 H34  *MINAP Variable: history of cerebrovascular disease* |
| Dementia | F00 F01 F02 F03 G30 F05 G31 |
| Chronic Pulmonary Disease | I27 J40 J41 J42 J43 J44 J45 J46 J47 J60 J61 J62 J63 J64 J65 J66 J67 J68 J70  *MINAP variable: history of asthma or COPD* |
| Connective Tissue Disease | M05 M06 M315 M32 M33 M34 M351 M353 M360 |
| Peptic Ulcer Disease | K25 K26 K27 K28 |
| Hemiplegia/Paraplegia | G81 G82 G83 |
| Diabetes without Complications | E10 E11 E12 E13 E14 E100 E101 E106 E108 E109 E110 E111 E116 E118 E119 E120 E121 E126 E128 E129 E130 E131 E136 E138 E139 E140 E141 E146 E148 E149  *MINAP variable: diabetes* |
| Diabetes with Complications | E102 E103 E104 E105 E107 E112 E115 E117 E122 E123 E124 E125 E127 E132 E133 E134 E135 E137 E142 E143 E144 E145 E147 |
| Mild Liver Disease | B18 K70 K73 K74 |
| Moderate/Severe Liver Disease | I850 I859 I864 I982 K704 K711 K721 K729 K765 K766 K767 |
| Renal Disease | I12 I13 N03 N05 N18 N19 N25 Z49 Z94 Z99  *MINAP variable: history of chronic kidney disease* |
| Any Malignancy | C00 C01 C02 C03 C04 C05 C06 C07 C08 C09 C10 C11 C12 C13 C14 C15 C16 C17 C18 C19 C20 C21 C22 C23 C24 C25 C26 C30 C31 C32 C33 C34 C37 C38 C39 C40 C41 C43 C45 C46 C47 C48 C49 C50 C51 C52 C53 C54 C55 C56 C57 C58 C60 C61 C62 C63 C64 C65 C66 C67 C68 C69 C70 C71 C72 C73 C74 C75 C76 C81 C82 C83 C84 C85 C88 C90 C91 C92 C93 C94 C95 C96 C97 |
| Metastatic Solid Tumor | C77 C78 C79 C80 |
| HIV/AIDS | B20 B21 B22 B24 |
