# Supplementary material for: Frailty and long-term outcomes in younger patients with acute myocardial infarction
Source: Eur Heart J. 2025 Nov 25;47(21):2686–96. doi: 10.1093/eurheartj/ehaf876 (PMC12766437; doi:10.1093/eurheartj/ehaf876)
Supplement: ehaf876_Supplementary_Data [file ehaf876_supplementary_data.zip › Supplementary Table 2 .docx]

**Supplementary Table 2**  ICD-10 codes used to extract outcomes from HES-APC and ONS mortality data.

| Neurological Haemorrhage | I60 I61 I62 S064 S065 S066 |
| --- | --- |
| Gastrointestinal Bleed | I850 I983 K226 K250 K252 K254 K256 K260 K262 K264 K266 K270 K272 K274 K276 K280 K282 K284 K286 K290 K625 K661 K762 K920 K921 K922 I848 I844 I841 |
| Ruptured Aortic Aneurysm | I713 I715 I711 I718 |
| Minor Bleed | R31X R58X H113 H356 H431 H450 H922 J942 M250 N939 N950 R040 R041 R042 R048 R049 N421 N021 N022 N023 N025 N028 I230 I312 S260 N836 N857 N897 N930 N939 N938 N920 N921 N924 |
| Ischaemic Stroke | I630 I631 I632 I633 I634 I635 I636 I638 I639 |
| Reinfarction Admission | I210 I211 I212 I213 I214 I219 I220 I221 I228 I229 |
| Heart Failure Admission | I110 I130 I132 I500 I501 I502 I503 I504 I508 I509 |
