# Supplementary material for: Frailty and long-term outcomes in younger patients with acute myocardial infarction
Source: Eur Heart J. 2025 Nov 25;47(21):2686–96. doi: 10.1093/eurheartj/ehaf876 (PMC12766437; doi:10.1093/eurheartj/ehaf876)
Supplement: ehaf876_Supplementary_Data [file ehaf876_supplementary_data.zip › Supplementary Table 3.docx]

| **Supplementary Table 3:** Clinical data and in-hospital treatments in patients with AMI stratified by age and frailty groups | | | | | | | | | | | | |
| --- | --- | --- | --- | --- | --- | --- | --- | --- | --- | --- | --- | --- |
|  | **Age Group (Years)** | | | | | | | | | | | |
|  | **<55 years** | | | | **55-74 years** | | | | **≥75 years** | | | |
|  | Fit | Mild Frailty | Moderate Frailty | Severe Frailty | Fit | Mild Frailty | Moderate Frailty | Severe Frailty | Fit | Mild Frailty | Moderate Frailty | Severe Frailty |
| N | 86,872 (55.5%) | 52,221 (33.3%) | 13,814 (8.8%) | 3,710 (2.4%) | 139,838 (34.1%) | 160,319 (39.1%) | 76,153 (18.6%) | 34,259 (8.3%) | 47,326 (13.0%) | 124,649 (34.3%) | 112,858 (31.0%) | 79,114 (21.7%) |
| ***Clinical characteristics*** | | | | | | | | | | | |  |
| *Left ventricular ejection fraction (echocardiogram)* | | | | | | | | | | | | |
| *Good (≥50%)* | 26,406 (69.9%) | 15,056 (60.2%) | 3,722 (49.4%) | 831 (37.4%) | 38,502 (66.7%) | 42,670 (59.2%) | 18,305 (48.5%) | 7,370 (38.7%) | 9,867 (57.9%) | 25,185 (52.9%) | 21,495 (45.3%) | 14,374 (38.4%) |
| *Moderate (30-49%)* | 10,032 (26.6%) | 8,078 (32.3%) | 2,769 (36.8%) | 923 (41.6%) | 16,670 (28.9%) | 23,300 (32.3%) | 13,703 (36.3%) | 7,360 (38.6%) | 5,750 (33.8%) | 16,572 (34.8%) | 17,500 (36.9%) | 14,421 (38.6%) |
| *Poor (<30%)* | 1,329 (3.5%) | 1,875 (7.5%) | 1,036 (13.8%) | 467 (21.0%) | 2,584 (4.5%) | 6,139 (8.5%) | 5,772 (15.3%) | 4,318 (22.7%) | 1,412 (8.3%) | 5,823 (12.2%) | 8,427 (17.8%) | 8,595 (23.0%) |
| Systolic blood pressure, median (IQR) | 137 (121-153) | 136 (120-155) | 134 (116-153) | 132 (111-154) | 140 (122-158) | 138 (120-157) | 136 (118-156) | 134 (114-155) | 140 (122-159) | 139 (120-159) | 136 (117-157) | 134 (114-155) |
| Heart rate, median (IQR) | 75 (65-87) | 78 (67-91) | 82 (70-96) | 86 (74-100) | 73 (63-85) | 76 (64-90) | 80 (67-96) | 84 (70-100) | 75 (64-88) | 78 (66-93) | 81 (68-97) | 83 (70-99) |
| Creatinine umol/L, median (IQR) | 80 (70-91) | 79 (68-92) | 81 (68-99) | 98 (75-161) | 82 (71-95) | 84 (71-100) | 90 (74-113) | 107 (82-155) | 89 (74-107) | 94 (77-117) | 102 (81-133) | 116 (88-158) |
| *Killip class* | | | | | | | | | | | | |
| *Killip class I* | 33,515 (87.3%) | 22,152 (75.6%) | 5,696 (60.7%) | 1,135 (39.8%) | 49,320 (78.2%) | 56,288 (61.9%) | 23,786 (45.0%) | 7,924 (28.9%) | 12,225 (54.6%) | 29,528 (39.2%) | 23,760 (29.2%) | 14,135 (21.8%) |
| *Killip class II* | 4,102 (10.7%) | 5,457 (18.6%) | 2,757 (29.4%) | 1,224 (42.9%) | 12,210 (19.4%) | 29,063 (32.0%) | 23,705 (44.9%) | 14,936 (54.4%) | 9,386 (41.9%) | 40,773 (54.1%) | 49,333 (60.6%) | 40,272 (62.2%) |
| *Killip class III* | 185 (0.5%) | 339 (1.2%) | 305 (3.3%) | 215 (7.5%) | 472 (0.7%) | 1,596 (1.8%) | 2,195 (4.2%) | 2,509 (9.1%) | 383 (1.7%) | 2,230 (3.0%) | 4,199 (5.2%) | 6,009 (9.3%) |
| *Killip class IV* | 576 (1.5%) | 1,351 (4.6%) | 625 (6.7%) | 277 (9.7%) | 1,051 (1.7%) | 3,919 (4.3%) | 3,153 (6.0%) | 2,071 (7.5%) | 388 (1.7%) | 2,862 (3.8%) | 4,159 (5.1%) | 4,310 (6.7%) |
| STEMI | 44,425 (51.1%) | 24,622 (47.1%) | 6,194 (44.8%) | 1,403 (37.8%) | 67,379 (48.2%) | 62,326 (38.9%) | 23,621 (31.0%) | 8,318 (24.3%) | 19,669 (41.6%) | 36,152 (29.0%) | 24,946 (22.1%) | 14,415 (18.2%) |
| NSTEMI | 42,447 (48.9%) | 27,599 (52.9%) | 7,620 (55.2%) | 2,307 (62.2%) | 72,459 (51.8%) | 97,993 (61.1%) | 52,532 (69.0%) | 25,941 (75.7%) | 27,657 (58.4%) | 88,497 (71.0%) | 87,912 (77.9%) | 64,699 (81.8%) |
| Cardiac arrest | 3,994 (4.8%) | 3,510 (7.0%) | 1,482 (11.1%) | 499 (13.9%) | 6,221 (4.6%) | 9,359 (6.1%) | 5,920 (8.1%) | 3,482 (10.5%) | 2,840 (6.2%) | 7,561 (6.3%) | 7,516 (6.9%) | 5,908 (7.7%) |
| ***Medications prescribed*** | | | | | | | | | | | |  |
| Dual antiplatelets | 71,836 (94.7%) | 42,582 (92.5%) | 11,115 (90%) | 2,860 (86.4%) | 113,540 (94.2%) | 126,505 (90.6%) | 57,627 (86.1%) | 24,755 (81.3%) | 34,528 (88.6%) | 87,917 (83.4%) | 75,896 (77.8%) | 50,182 (72.4%) |
| Fondaparinux or LMWH | 48,439 (67.5%) | 29,473 (68.7%) | 7,717 (67.7%) | 2,083 (68.3%) | 82,083 (70.8%) | 97,617 (73.1%) | 47,259 (73.6%) | 21,247 (73.4%) | 28,518 (73.4%) | 80,795 (76.3%) | 74,705 (76.6%) | 51,660 (74.9%) |
| Unfractionated heparin | 24,366 (34.8%) | 13,481 (32.1%) | 3,487 (31.1%) | 872 (29.3%) | 37,341 (33.3%) | 36,903 (28.5%) | 15,318 (24.5%) | 6,284 (22.1%) | 9,303 (24.8%) | 18,944 (18.5%) | 14,066 (14.8%) | 8,745 (12.9%) |
| Warfarin | 851 (1.2%) | 964 (2.3%) | 475 (4.3%) | 193 (6.5%) | 1,929 (1.7%) | 4,767 (3.7%) | 4,874 (7.8%) | 3,109 (10.9%) | 1,186 (3.2%) | 6,358 (6.2%) | 9,006 (9.4%) | 7,827 (11.5%) |
| Glycoprotein IIb/IIIa inhibitors | 9,988 (14.0%) | 5,308 (12.4%) | 1,343 (11.8%) | 299 (9.9%) | 13,724 (12.0%) | 12,893 (9.7%) | 4,811 (7.6%) | 1,658 (5.8%) | 2,807 (7.3%) | 5,259 (5.0%) | 3,433 (3.6%) | 1,674 (2.4%) |
| ACE inhibitor or ARB | 64,639 (89.1%) | 40,592 (89.9%) | 10,691 (88.5%) | 2,598 (81.5%) | 103,823 (89.2%) | 121,610 (88.0%) | 56,769 (85.3%) | 23,301 (78.6%) | 31,886 (81.8%) | 83,331 (78.5%) | 72,262 (74.3%) | 45,618 (68.2%) |
| Beta-blocker | 70,822 (91.3%) | 41,968 (89.1%) | 10,891 (87.5%) | 2,854 (86.1%) | 112,299 (90.2%) | 123,005 (85.5%) | 55,879 (81.7%) | 24,814 (81.1%) | 34,405 (83.6%) | 86,477 (78.5%) | 75,627 (75.5%) | 52,767 (75.8%) |
| High dose statin | 71,221 (96.7%) | 43,992 (96.1%) | 11,695 (95.4%) | 3,069 (93.2%) | 115,560 (97.1%) | 135,527 (95.6%) | 64,394 (94.1%) | 28,627 (92.8%) | 36,795 (92.7%) | 97,295 (89.9%) | 86,619 (87.5%) | 58,866 (85.4%) |
| Mineralocorticoid receptor antagonist | 2,623 (5.7%) | 2,920 (9.6%) | 1,391 (15.7%) | 498 (20.3%) | 4,278 (6.2%) | 8,231 (9.6%) | 6,691 (15.2%) | 4,571 (21.4%) | 1,475 (7.1%) | 5,621 (9.3%) | 8,089 (13.2%) | 7,903 (16.5%) |
| ***Interventional management*** | | | | | | | | | | | |  |
| Invasive coronary angiogram | 54,356 (75.1%) | 33,195 (76.6%) | 8,776 (77.6%) | 2,130 (74.4%) | 83,227 (71.5%) | 90,414 (69.3%) | 38,564 (65.2%) | 14,868 (60.5%) | 19,592 (51.6%) | 42,236 (44.4%) | 30,812 (38.7%) | 16,912 (35.0%) |
| PCI | 49,080 (67.8%) | 29,240 (67.5%) | 7,432 (65.8%) | 1,698 (59.3%) | 72,683 (62.5%) | 75,425 (57.8%) | 30,242 (51.1%) | 11,118 (45.2%) | 16,535 (43.6%) | 33,744 (35.4%) | 23,708 (29.8%) | 12,778 (26.4%) |
| CABG surgery | 1,124 (1.6%) | 1,015 (2.4%) | 370 (3.3%) | 152 (5.5%) | 3,157 (2.7%) | 4,632 (3.6%) | 2,509 (4.3%) | 1,084 (4.5%) | 781 (2.1%) | 2,023 (2.2%) | 1,439 (1.8%) | 793 (1.7%) |
| Abbreviations: FI = frailty index, IQR = interquartile range, STEMI = ST-elevation myocardial infarction, NSTEMI = non ST-elevation myocardial infarction, LMWH = low molecular weight heparin, ACE = angiotensin converting enzyme, ARB = angiotensin receptor blocker, PCI = percutaneous coronary intervention, CABG = coronary artery bypass graft | | | | | | | | | | | | |
