# Supplementary material for: Frailty and long-term outcomes in younger patients with acute myocardial infarction
Source: Eur Heart J. 2025 Nov 25;47(21):2686–96. doi: 10.1093/eurheartj/ehaf876 (PMC12766437; doi:10.1093/eurheartj/ehaf876)
Supplement: ehaf876_Supplementary_Data [file ehaf876_supplementary_data.zip › Supplementary Table 5.docx]

| **Supplementary Table 5 :** Summary of short-term outcomes (30 days) in patients with AMI stratified by age and frailty groups | | | | |
| --- | --- | --- | --- | --- |
|  |  | **Age Category** | | |
|  |  | **<55 years** | **55-74 years** | **≥75 years** |
| **Outcome** | **SCARF Index Category** | Adjusted Odds Ratio (95% Confidence Interval) | Adjusted Odds Ratio (95% Confidence Interval) | Adjusted Odds Ratio (95% Confidence Interval) |
| All-Cause Death | Fit | 1(ref) | 1(ref) | 1(ref) |
|  | Mild | 1.39 (1.25-1.55) | 1.28 (1.22-1.35) | 1.20 (1.15-1.26) |
|  | Moderate | 1.65 (1.44-1.90) | 1.71 (1.62-1.81) | 1.42 (1.35-1.48) |
|  | Severe | 2.46 (2.05-2.95) | 2.31 (2.16-2.47) | 1.73 (1.65-1.82) |
| Cardiovascular Death | Fit | 1(ref) | 1(ref) | 1(ref) |
|  | Mild | 1.32 (1.16-1.49) | 1.09 (1.03-1.16) | 1.03 (0.98-1.08) |
|  | Moderate | 1.48 (1.26-1.74) | 1.32 (1.23-1.41) | 1.10 (1.04-1.16) |
|  | Severe | 1.85 (1.49-2.30) | 1.59 (1.47-1.72) | 1.17 (1.11-1.23) |
| MACE | Fit | 1(ref) | 1(ref) | 1(ref) |
|  | Mild | 1.16 (1.10-1.22) | 1.19 (1.16-1.23) | 1.17 (1.13-1.21) |
|  | Moderate | 1.59 (1.48-1.72) | 1.56 (1.51-1.62) | 1.41 (1.36-1.46) |
|  | Severe | 2.36 (2.12-2.64) | 2.13 (2.04-2.22) | 1.72 (1.65-1.78) |
| Heart Failure Readmission | Fit | 1(ref) | 1(ref) | 1(ref) |
|  | Mild | 1.75 (1.55-1.97) | 1.83 (1.71-1.96) | 1.37 (1.28-1.46) |
|  | Moderate | 3.34 (2.90-3.86) | 3.00 (2.80-3.22) | 1.98 (1.85-2.12) |
|  | Severe | 5.27 (4.38-6.34) | 4.59 (4.24-4.97) | 2.60 (2.43-2.79) |
| Reinfarction | Fit | 1(ref) | 1(ref) | 1(ref) |
|  | Mild | 1.07 (1.01-1.13) | 1.07 (1.03-1.11) | 1.11 (1.05-1.17) |
|  | Moderate | 1.39 (1.27-1.53) | 1.21 (1.15-1.27) | 1.16 (1.10-1.22) |
|  | Severe | 1.45 (1.23-1.70) | 1.28 (1.20-1.37) | 1.13 (1.07-1.20) |
| Major Bleed | Fit | 1(ref) | 1(ref) | 1(ref) |
|  | Mild | 1.53 (1.31-1.78) | 1.34 (1.24-1.46) | 1.13 (1.04-1.24) |
|  | Moderate | 1.85 (1.49-2.29) | 1.83 (1.67-2.02) | 1.34 (1.22-1.46) |
|  | Severe | 2.17 (1.58-2.97) | 2.23 (1.99-2.50) | 1.58 (1.43-1.74) |
| Minor Bleed | Fit | 1(ref) | 1(ref) | 1(ref) |
|  | Mild | 1.17 (1.00-1.35) | 1.17 (1.08-1.27) | 1.28 (1.16-1.41) |
|  | Moderate | 1.31 (1.04-1.63) | 1.42 (1.28-1.57) | 1.49 (1.34-1.65) |
|  | Severe | 1.42 (1.00-2.00) | 1.95 (1.72-2.21) | 1.84 (1.65-2.05) |
