# Supplementary material for: Frailty and long-term outcomes in younger patients with acute myocardial infarction
Source: Eur Heart J. 2025 Nov 25;47(21):2686–96. doi: 10.1093/eurheartj/ehaf876 (PMC12766437; doi:10.1093/eurheartj/ehaf876)
Supplement: ehaf876_Supplementary_Data [file ehaf876_supplementary_data.zip › Supplementary Table 6.docx]

| **Supplementary Table 6 :** Relative odds ratios of 30 day outcomes in patients with AMI (with patients aged 75 and older used as reference group) | | | | | |
| --- | --- | --- | --- | --- | --- |
|  | **Age Category (Years)** | | | | |
|  |  | **<55 years** | | **55-74 years** | |
| **Outcome** | **SCARF Index Category** | **Relative Odds Ratio (95% Confidence Interval)** | **P Interaction** | **Relative Odds Ratio (95% Confidence Interval)** | **P Interaction** |
| All-Cause Death | Mild | 1.28 (1.14-1.42) | <0.001 | 1.13 (1.06-1.21) | <0.001 |
|  | Moderate | 1.39 (1.22-1.58) | <0.001 | 1.33 (1.25-1.43) | <0.001 |
|  | Severe | 1.70 (1.45-2.00) | <0.001 | 1.50 (1.39-1.60) | <0.001 |
| Cardiovascular Death | Mild | 1.41 (1.24-1.60) | <0.001 | 1.12 (1.04-1.21) | 0.003 |
|  | Moderate | 1.61 (1.38-1.87) | <0.001 | 1.32 (1.22-1.42) | <0.001 |
|  | Severe | 1.89 (1.56-2.29) | <0.001 | 1.50 (1.39-1.63) | <0.001 |
| MACE | Mild | 0.91 (0.86-0.97) | 0.004 | 1.03 (0.99-1.08) | 0.164 |
|  | Moderate | 0.95 (0.88-1.03) | 0.224 | 1.12 (1.07-1.18) | <0.001 |
|  | Severe | 1.06 (0.95-1.18) | 0.283 | 1.24 (1.18-1.30) | <0.001 |
| Heart Failure Readmission | Mild | 1.33 (1.17-1.52) | <0.001 | 1.40 (1.27-1.53) | <0.001 |
|  | Moderate | 1.87 (1.63-2.16) | <0.001 | 1.65 (1.51-1.81) | <0.001 |
|  | Severe | 2.31 (1.95-2.72) | <0.001 | 1.99 (1.81-2.18) | <0.001 |
| Reinfarction | Mild | 0.92 (0.85-0.99) | 0.028 | 0.96 (0.90-1.02) | 0.157 |
|  | Moderate | 1.12 (1.01-1.23) | 0.025 | 1.05 (0.98-1.12) | 0.175 |
|  | Severe | 1.17 (1.00-1.37) | 0.053 | 1.14 (1.05-1.23) | 0.001 |
| Major Bleed | Mild | 1.51 (1.27-1.78) | <0.001 | 1.31 (1.16-1.47) | <0.001 |
|  | Moderate | 1.82 (1.47-2.24) | <0.001 | 1.68 (1.49-1.89) | <0.001 |
|  | Severe | 2.22 (1.67-2.95) | <0.001 | 1.89 (1.66-2.15) | <0.001 |
| Minor Bleed | Mild | 1.05 (0.88-1.24) | 0.604 | 0.99 (0.88-1.13) | 0.927 |
|  | Moderate | 1.20 (0.96-1.50) | 0.117 | 1.11 (0.98-1.27) | 0.109 |
|  | Severe | 1.29 (0.94-1.77) | 0.115 | 1.31 (1.14-1.51) | <0.001 |
