# Supplementary material for: Frailty and long-term outcomes in younger patients with acute myocardial infarction
Source: Eur Heart J. 2025 Nov 25;47(21):2686–96. doi: 10.1093/eurheartj/ehaf876 (PMC12766437; doi:10.1093/eurheartj/ehaf876)
Supplement: ehaf876_Supplementary_Data [file ehaf876_supplementary_data.zip › Supplementary Table 7.docx]

| **Supplementary Table 7:** Crude outcomes by age and frailty status | | | | |
| --- | --- | --- | --- | --- |
|  | Fit | Mild Frailty | Moderate Frailty | Severe Frailty |
| **Age <55 years** | | | | |
| Length of hospital stay (days), median (IQR) | 3 (2-5) | 3 (2-5) | 4 (2-7) | 6 (3-12) |
| In-hospital death | 578 (0.7%) | 786 (1.5%) | 426 (3.1%) | 258 (7.0%) |
| In-hospital reinfarction | 910 (1.2%) | 582 (1.3%) | 219 (1.8%) | 54 (1.6%) |
| In-hospital major bleeding | 246 (0.3%) | 251 (0.5%) | 111 (0.9%) | 44 (1.3%) |
| In-hospital minor bleeding | 312 (0.4%) | 228 (0.5%) | 79 (0.6%) | 29 (0.8%) |
| All-cause death (30 days) | 826 (1.0%) | 1,043 (2.0%) | 561 (4.1%) | 329 (8.9%) |
| Cardiovascular death (30 days) | 603 (0.7%) | 748 (1.4%) | 389 (2.8%) | 204 (5.5%) |
| MACE (30 days) | 3,997 (4.6%) | 3,127 (6.0%) | 1,385 (10.0%) | 675 (18.2%) |
| Reinfarction (30 days) | 3,588 (4.1%) | 2,107 (4.0%) | 686 (5.0%) | 195 (5.3%) |
| Heart failure admission (30 days) | 546 (0.6%) | 704 (1.3%) | 473 (3.4%) | 272 (7.3%) |
| Major bleeding (30 days) | 375 (0.4%) | 371 (0.7%) | 147 (1.1%) | 63 (1.7%) |
| Minor bleeding (30 days) | 430 (0.5%) | 338 (0.6%) | 122 (0.9%) | 48 (1.3%) |
| All-cause death (1 year)* | 784 (0.9%) | 914 (1.8%) | 590 (4.4%) | 444 (12.9%) |
| Cardiovascular death (1 year)* | 288 (0.3%) | 336 (0.7%) | 221 (1.7%) | 155 (4.5%) |
| MACE (1 year)* | 6,406 (7.4%) | 5,564 (10.7%) | 2,633 (19.1%) | 1,346 (36.3%) |
| Reinfarction admission (1 year)* | 4,047 (4.7%) | 2,680 (5.2%) | 989 (7.4%) | 379 (11.0%) |
| Heart failure admission (1 year)* | 1,929 (2.2%) | 2,514 (4.9%) | 1,540 (11.5%) | 907 (26.3%) |
| Major bleeding (1 year)* | 1,164 (1.3%) | 824 (1.6%) | 296 (2.2%) | 135 (3.9%) |
| Minor bleeding (1 year)* | 893 (1.0%) | 762 (1.5%) | 325 (2.4%) | 138 (4.0%) |
| **Age 55-74 years** | | | | |
| Length of hospital stay (days), median (IQR) | 4 (2-6) | 4 (2-7) | 5 (3-9) | 7 (4-14) |
| In-hospital death | 2,398 (1.7%) | 4,658 (2.9%) | 4,148 (5.4%) | 3,566 (10.4%) |
| In-hospital reinfarction | 1,628 (1.3%) | 1,859 (1.3%) | 1,028 (1.5%) | 545 (1.8%) |
| In-hospital major bleeding | 605 (0.5%) | 987 (0.6%) | 723 (1.0%) | 497 (1.5%) |
| In-hospital minor bleeding | 717 (0.5%) | 976 (0.6%) | 611 (0.8%) | 419 (1.3%) |
| All-cause death (30 days) | 3,218 (2.3%) | 6,151 (3.8%) | 5,283 (6.9%) | 4,250 (12.4%) |
| Cardiovascular death (30 days) | 2,449 (1.8%) | 4,143 (2.6%) | 3,314 (4.4%) | 2,493 (7.3%) |
| MACE (30 days) | 8,718 (6.2%) | 13,928 (8.7%) | 10,518 (13.8%) | 7,713 (22.5%) |
| Reinfarction (30 days) | 6,071 (4.3%) | 7,083 (4.4%) | 3,767 (4.9%) | 1,815 (5.3%) |
| Heart failure admission (30 days) | 1,306 (0.9%) | 3,348 (2.1%) | 3,337 (4.4%) | 2,876 (8.4%) |
| Major bleeding (30 days) | 985 (0.7%) | 1,620 (1.0%) | 1,191 (1.6%) | 768 (2.2%) |
| Minor bleeding (30 days) | 1,031 (0.7%) | 1,472 (0.9%) | 926 (1.2%) | 638 (1.9%) |
| All-cause death (1 year)* | 3,094 (2.3%) | 7,511 (4.8%) | 7,252 (10.1%) | 6,100 (19.9%) |
| Cardiovascular death (1 year)* | 910 (0.7%) | 2,102 (1.4%) | 1,937 (2.7%) | 1,554 (5.1%) |
| MACE (1 year)* | 13,247 (9.5%) | 24,508 (15.3%) | 19,743 (25.9%) | 13,995 (40.9%) |
| Reinfarction admission (1 year)* | 6,719 (4.9%) | 9,284 (6.0%) | 5,872 (8.2%) | 3,327 (10.8%) |
| Heart failure admission (1 year)* | 4,581 (3.3%) | 11,624 (7.5%) | 11,540 (16.0%) | 9,312 (30.3%) |
| Major bleeding (1 year)* | 2,384 (1.7%) | 3,736 (2.4%) | 2,426 (3.4%) | 1,379 (4.5%) |
| Minor bleeding (1 year)* | 2,171 (1.6%) | 3,229 (2.1%) | 1,992 (2.8%) | 1,203 (3.9%) |
| **Age ≥75 years** | | | | |
| Length of hospital stay (days), median (IQR) | 5 (3-8) | 5 (3-10) | 7 (4-12) | 9 (5-16) |
| In-hospital death | 3,422 (7.2%) | 11,581 (9.3%) | 14,254 (12.6%) | 14,293 (18.1%) |
| In-hospital reinfarction | 602 (1.5%) | 1,765 (1.6%) | 1,577 (1.6%) | 1,195 (1.7%) |
| In-hospital major bleeding | 422 (0.9%) | 1,167 (1.0%) | 1,272 (1.2%) | 1,105 (1.5%) |
| In-hospital minor bleeding | 338 (0.8%) | 1,134 (1.0%) | 1,248 (1.2%) | 1,121 (1.5%) |
| All-cause death (30 days) | 4,118 (8.7%) | 13,867 (11.1%) | 16,792 (14.9%) | 16,078 (20.3%) |
| Cardiovascular death (30 days) | 3,168 (6.7%) | 9,391 (7.5%) | 10,528 (9.3%) | 9,287 (11.7%) |
| MACE (30 days) | 6,591 (13.9%) | 21,819 (17.5%) | 26,130 (23.2%) | 24,185 (30.6%) |
| Reinfarction (30 days) | 2,249 (4.8%) | 6,332 (5.1%) | 5,843 (5.2%) | 3,919 (5.0%) |
| Heart failure admission (30 days) | 1,164 (2.5%) | 4,807 (3.9%) | 7,167 (6.4%) | 7,270 (9.2%) |
| Major bleeding (30 days) | 716 (1.5%) | 2,085 (1.7%) | 2,262 (2.0%) | 1,929 (2.4%) |
| Minor bleeding (30 days) | 540 (1.1%) | 1,785 (1.4%) | 1,900 (1.7%) | 1,673 (2.1%) |
| All-cause death (1 year)* | 4,558 (10.4%) | 18,775 (16.6%) | 24,807 (25.2%) | 22,746 (35.1%) |
| Cardiovascular death (1 year)* | 1,151 (2.6%) | 4,535 (4.0%) | 5,686 (5.8%) | 4,962 (7.7%) |
| MACE (1 year)* | 9,132 (19.3%) | 35,184 (28.2%) | 43,329 (38.4%) | 37,013 (46.8%) |
| Reinfarction admission (1 year)* | 3,057 (7.0%) | 10,300 (9.1%) | 10,722 (10.9%) | 7,227 (11.1%) |
| Heart failure admission (1 year)* | 3,617 (8.2%) | 16,440 (14.5%) | 23,533 (23.9%) | 21,983 (33.9%) |
| Major bleeding (1 year)* | 1,494 (3.4%) | 4,535 (4.0%) | 4,805 (4.9%) | 3,592 (5.5%) |
| Minor bleeding (1 year)* | 1,238 (2.8%) | 3,660 (3.2%) | 3,609 (3.7%) | 2,586 (4.0%) |
| *In-hospital events excluded | | | | |
