# Supplementary material for: Frailty and long-term outcomes in younger patients with acute myocardial infarction
Source: Eur Heart J. 2025 Nov 25;47(21):2686–96. doi: 10.1093/eurheartj/ehaf876 (PMC12766437; doi:10.1093/eurheartj/ehaf876)
Supplement: ehaf876_Supplementary_Data [file ehaf876_supplementary_data.zip › Supplementary Table_4.docx]

| **Supplementary Table 4:** Summary of age and frailty interaction analysis, as continuous variables | | |
| --- | --- | --- |
| **1-year outcomes** | | |
| **Outcome** | **Adjusted Hazard Ratio (95% CI)** | **p interaction value** |
| All-Cause Death | 0.994 (0.994–0.994) | <0.001 |
| Cardiovascular Death | 0.994 (0.994–0.995) | <0.001 |
| MACE | 0.997 (0.997–0.997) | <0.001 |
| Heart Failure | 0.995 (0.995–0.995) | <0.001 |
| Reinfarction | 0.999 (0.998–0.999) | <0.001 |
| Major Bleed | 0.997 (0.997–0.998) | <0.001 |
| Minor Bleed | 0.996 (0.996–0.997) | <0.001 |
| **30-day outcomes** | | |
| **Outcome** | **Adjusted Odds Ratio (95% CI)** | **p interaction value** |
| All-Cause Death | 0.997 (0.997–0.998) | <0.001 |
| Cardiovascular Death | 0.997 (0.997–0.998) | <0.001 |
| MACE | 1.000 (0.999–1.000) | 0.002 |
| Heart Failure | 0.994 (0.994–0.995) | <0.001 |
| Reinfarction | 0.999 (0.998–0.999) | <0.001 |
| Major Bleed | 0.995 (0.994–0.996) | <0.001 |
| Minor Bleed | 0.998 (0.997–0.998) | <0.001 |
