# Supplementary material for: Frailty and long-term outcomes in younger patients with acute myocardial infarction
Source: Eur Heart J. 2025 Nov 25;47(21):2686–96. doi: 10.1093/eurheartj/ehaf876 (PMC12766437; doi:10.1093/eurheartj/ehaf876)
Supplement: ehaf876_Supplementary_Data [file ehaf876_supplementary_data.zip › Supp_Fig_2.pdf]

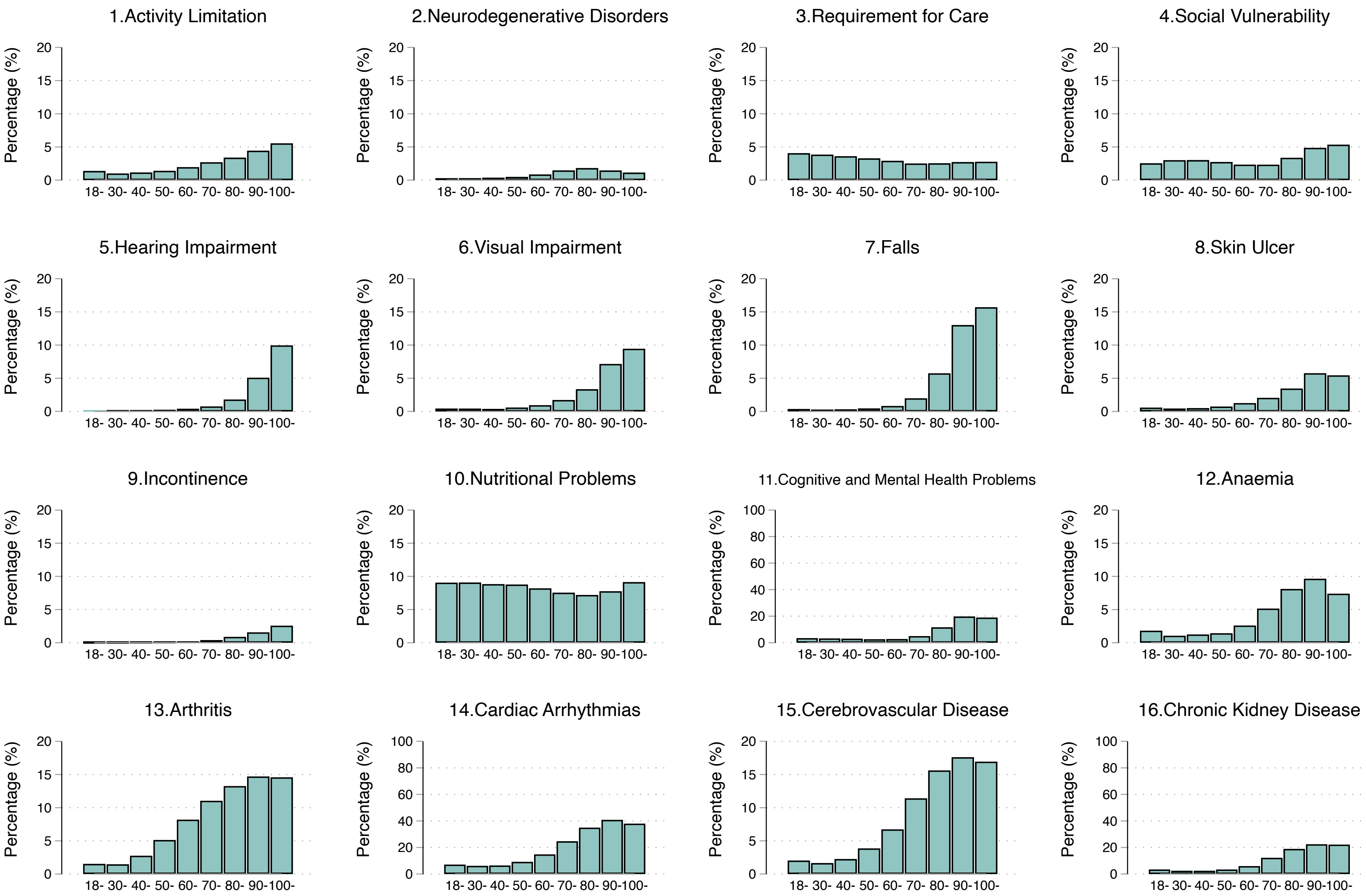

Supplementary Figure 2: Prevalence of SCARF index health deficits by age

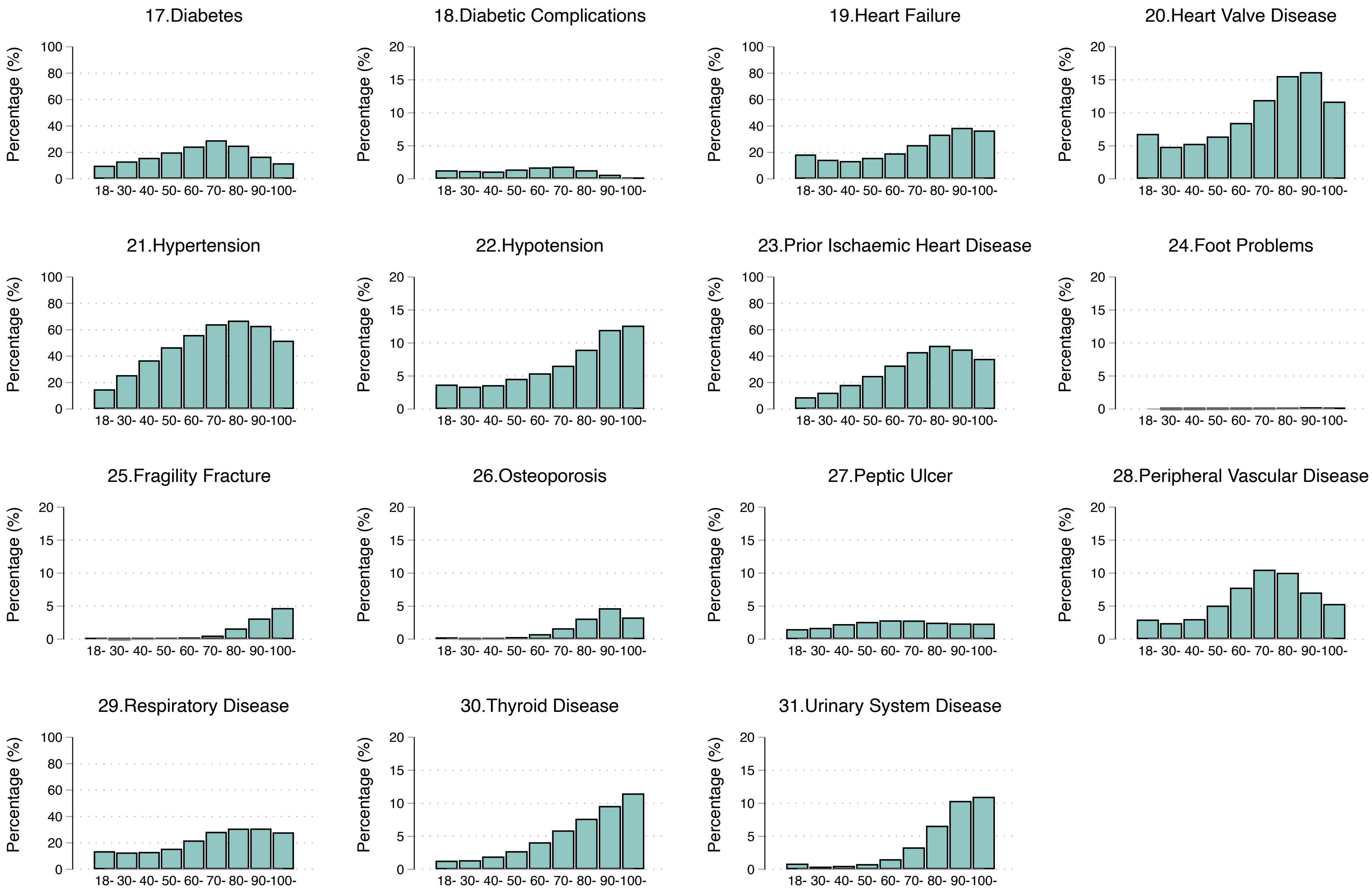

Supplementary Figure 2 continued: Prevalence of SCARF index health deficits by age
